# Overweight, obesity, and cardiovascular disease in heterozygous familial hypercholesterolaemia: the EAS FH Studies Collaboration registry

**DOI:** 10.1093/eurheartj/ehae791

**Published:** 2025-01-13

**Authors:** Amany Elshorbagy, Antonio J Vallejo-Vaz, Fotios Barkas, Alexander R M Lyons, Christophe A T Stevens, Kanika I Dharmayat, Alberico L Catapano, Tomas Freiberger, G Kees Hovingh, Pedro Mata, Frederick J Raal, Raul D Santos, Handrean Soran, Gerald F Watts, Marianne Abifadel, Carlos A Aguilar-Salinas, Khalid F Alhabib, Mutaz Alkhnifsawi, Wael Almahmeed, Fahad Alnouri, Rodrigo Alonso, Khalid Al-Rasadi, Ahmad Al-Sarraf, Marcello Arca, Tester F Ashavaid, Maurizio Averna, Maciej Banach, Marianne Becker, Christoph J Binder, Mafalda Bourbon, Liam R Brunham, Krzysztof Chlebus, Pablo Corral, Diogo Cruz, Kairat Davletov, Olivier S Descamps, Bambang Dwiputra, Marat Ezhov, Urh Groselj, Mariko Harada-Shiba, Kirsten B Holven, Steve E Humphries, Meral Kayikcioglu, Weerapan Khovidhunkit, Katarina Lalic, Gustavs Latkovskis, Ulrich Laufs, Evangelos Liberopoulos, Marcos M Lima-Martinez, Vincent Maher, A David Marais, Winfried März, Erkin Mirrakhimov, André R Miserez, Olena Mitchenko, Hapizah Nawawi, Børge G Nordestgaard, Andrie G Panayiotou, György Paragh, Zaneta Petrulioniene, Belma Pojskic, Arman Postadzhiyan, Ashraf Reda, Željko Reiner, Ximena Reyes, Fouzia Sadiq, Wilson Ehidiamen Sadoh, Heribert Schunkert, Aleksandr B Shek, Erik Stroes, Ta-Chen Su, Tavintharan Subramaniam, Andrey V Susekov, Myra Tilney, Brian Tomlinson, Thanh Huong Truong, Alexandros D Tselepis, Anne Tybjærg-Hansen, Alejandra Vázquez-Cárdenas, Margus Viigimaa, Branislav Vohnout, Shizuya Yamashita, Kausik K Ray

**Affiliations:** Department of Primary Care and Public Health, Imperial Centre for Cardiovascular Disease Prevention, School of Public Health, Imperial College London, White City Campus, 90 Wood Lane, London W12 0BZ, UK; Department of Physiology, Faculty of Medicine, University of Alexandria, Mowassat Campus, Alexandria, Egypt; Department of Primary Care and Public Health, Imperial Centre for Cardiovascular Disease Prevention, School of Public Health, Imperial College London, White City Campus, 90 Wood Lane, London W12 0BZ, UK; Clinical Epidemiology and Vascular Risk, Instituto de Biomedicina de Sevilla, IBiS/Hospital Universitario Virgen del Rocío/Universidad de Sevilla/CSIC, Seville, Spain; Faculty of Medicine, Department of Medicine, University of Seville, Seville, Spain; Centro de Investigación Biomédica en Red (CIBER) de Epidemiología y Salud Pública, Instituto de Salud Carlos III, Madrid, Spain; Department of Primary Care and Public Health, Imperial Centre for Cardiovascular Disease Prevention, School of Public Health, Imperial College London, White City Campus, 90 Wood Lane, London W12 0BZ, UK; Faculty of Medicine, Department of Internal Medicine, School of Health Sciences, University of Ioannina, Ioannina, Greece; Department of Primary Care and Public Health, Imperial Centre for Cardiovascular Disease Prevention, School of Public Health, Imperial College London, White City Campus, 90 Wood Lane, London W12 0BZ, UK; Department of Primary Care and Public Health, Imperial Centre for Cardiovascular Disease Prevention, School of Public Health, Imperial College London, White City Campus, 90 Wood Lane, London W12 0BZ, UK; Department of Primary Care and Public Health, Imperial Centre for Cardiovascular Disease Prevention, School of Public Health, Imperial College London, White City Campus, 90 Wood Lane, London W12 0BZ, UK; Department of Pharmacological and Biomolecular Sciences, University of Milan, Milan, Italy; Istituto di Ricovero e Cura a Carattere Scientifico (IRCCS) MultiMedica, Milan, Italy; Centre for Cardiovascular Surgery and Transplantation, and Medical Faculty, Masaryk University, Brno, Czech Republic; CarDia—National Institute for Metabolic and Cardiovascular Disease Research, Czech Republic; Department of Vascular Medicine, Amsterdam UMC, University of Amsterdam, Amsterdam, The Netherlands; Novo Nordisk, Soborg, Denmark; Fundación Hipercolesterolemia Familiar, Madrid, Spain; Carbohydrate and Lipid Metabolism Research Unit, Faculty of Health Sciences, University of the Witwatersrand, Johannesburg, South Africa; Heart Institute (InCor), University of São Paulo and Hospital Israelita Albert Einstein, São Paulo, Brazil; Manchester University NHS Foundation Trust, Manchester, UK; Faculty of Health and Medical Sciences, School of Medicine, University of Western Australia, Perth, WA, Australia; Department of Cardiology, Lipid Disorders Clinic, Cardiometabolic Services, Royal Perth Hospital, Perth, WA, Australia; Faculty of Pharmacy, Laboratory of Biochemistry and Molecular Therapeutics, Saint Joseph University, Beirut, Lebanon; Unidad de Investigación de Enfermedades Metabólicas, Instituto Nacional de Ciencias Médicas y Nutrición Salvador Zubirán, Mexico City, México; Tecnológico de Monterrey, Escuela de Medicina y Ciencias de la Salud, Monterrey, Mexico; Department of Cardiac Sciences, King Fahad Cardiac Centre, College of Medicine, King Saud University Medical City, King Saud University, Riyadh, Saudi Arabia; College of Pharmacy, University of Al Qadisiyah, Al Diwaniyah, Iraq; College of Medicine, University of Warith Al-Anbiyaa, Karbala, Iraq; Cleveland Clinic Abu Dhabi, Heart and Vascular Institute, Abu Dhabi, United Arab Emirates; Cardiovascular Prevention Unit, Adult Cardiology Department, Prince Sultan Cardiac Centre, Riyadh, Saudi Arabia; Centre for Advanced Metabolic Medicine and Nutrition, Santiago, Chile; Department of Biochemistry, College of Medicine and Health Science and Medical Research Centre, Sultan Qaboos University, Muscat, Oman; Sabah Al Ahmad Cardiac Centre, Kuwait City, Kuwait; Department of Translational and Precision Medicine, Sapienza University of Rome, Rome, Italy; Department of Laboratory Medicine, PD Hinduja Hospital and Medical Research Centre Mahim, Mumbai, India; Department of Health Promotion Sciences, Maternal and Infantile Care, Internal Medicine and Medical Specialties, University of Palermo, Palermo, Italy; Istituto di Biofisica, Consiglio Nazionale delle Ricerche, Palermo, Italy; Department of Preventive Cardiology and Lipidology, Medical University of Lodz (MUL), Lodz, Poland; Department of Cardiology and Congenital Diseases of Adults, Polish Mother’s Memorial Hospital Research Institute (PMMHRI), Lodz, Poland; Cardiovascular Research Centre, University of Zielona Gora, Zielona Gora, Poland; Department of Pediatric Endocrinology and Diabetology, Centre hospitalier de Luxembourg, Luxembourg City, Luxembourg; Department of Laboratory Medicine, Medical University of Vienna, Vienna, Austria; Unidade de Investigação e Desenvolvimento, Grupo de Investigação Cardiovascular, Departamento de Promoção da Saúde e Prevenção de Doenças Não Transmissíveis, Instituto Nacional de Saúde Doutor Ricardo Jorge, Lisbon, Portugal; Faculty of Sciences, Biosystems and Integrative Sciences Institute (BioISI), University of Lisbon, Lisbon, Portugal; Departments of Medicine and Medical Genetics, Centre for Heart Lung Innovation, The University of British Columbia, Vancouver, Canada; 1st Department of Cardiology Medical University of Gdańsk, National Centre of Familial Hypercholesterolaemia in Gdańsk, Gdańsk, Poland; Pharmacology Department, FASTA University, School of Medicine, Mar del Plata, Argentina; Portuguese Atherosclerosis Society, Lisbon, Portugal; Research Health Institute, Al Farabi Kazakh National University, Almaty, Kazakhstan; Centres Hospitaliers Universitaires HELORA at La Louvière and University of Mons, Mons, Belgium; Faculty of Medicine, Department of Cardiology and Vascular Medicine, Universitas Indonesia—Harapan Kita National Cardiovascular Center, Jakarta, Indonesia; National Medical Research Centre of Cardiology of Ministry of Health of the Russian Federation, Moscow, Russia; Department of Paediatric Endocrinology, Diabetes and Metabolism, University Medical Centre, University Children’s Hospital Ljubljana, Ljubljana, Slovenia; Faculty of Medicine, University of Ljubljana, Ljubljana, Slovenia; Osaka Medical and Pharmaceutical University, Takatsuki, Japan; National Advisory Unit on Familial Hypercholesterolemia, Oslo University Hospital, Oslo, Norway; Centre for Cardiovascular Genetics, Institute for Cardiovascular Science, University College London, London, UK; Department of Cardiology, Ege University Medical School, İzmir, Turkey; Faculty of Medicine, Department of Medicine, Chulalongkorn University and King Chulalongkorn Memorial Hospital, Bangkok, Thailand; Faculty of Medicine, Clinic for Endocrinology, Diabetes and Metabolic Diseases, University of Belgrade, Belgrade, Serbia; Faculty of Medicine, Research Institute of Cardiology and Regenerative Medicine, University of Latvia, Pauls Stradins Clinical University Hospital, Riga, Latvia; Klinik und Poliklinik für Kardiologie, Universitätsklinikum Leipzig, Leipzig, Germany; First Department of Propaedeutic and Internal Medicine, School of Medicine, National and Kapodistian University of Athens, Athens, Greece; Universidad de Oriente, Núcleo Bolívar, Ciudad Bolívar, Venezuela; Advanced Lipid Management and Research Centre (ALMAR), Tallaght University Hospital, Dublin, Ireland; Chemical Pathology, University of Cape Town Health Science Faculty, Cape Town, South Africa; DACH Society for the Prevention of Heart and Circulatory Diseases, Hamburg, Germany; Medical Faculty Mannheim, Department of Internal Medicine, Heidelberg University, Mannheim, Germany; Klinisches Institut für Medizinische und Chemische Labordiagnostik, Medizinische Universität Graz, Graz, Austria; Synlab Akademie, Synlab Holding Deutschland, Mannheim and Augsburg, Germany; Kyrgyz State Medical Academy, Bishkek, Kyrgyzstan; College of Medicine, Korea University, Seoul, Korea; Diagene Research Institute and Swiss Society for Familial Forms of Hypercholesterolemia (SSFH), Reinach, Switzerland; Faculty of Medicine, University of Basel, Basel, Switzerland; Department of Dyslipidaemia, Institute of Cardiology, National Academy of Medical Sciences, Kiev, Ukraine; Institute of Pathology, Laboratory and Forensic Medicine (I-PPerForM) and Faculty of Medicine, Universiti Teknologi MARA (UiTM), Sungai Buloh, Selangor, Malaysia; Herlev and Gentofte Hospital, Copenhagen University Hospital, University of Copenhagen, Copenhagen, Denmark; Department of Rehabilitation Sciences, School of Health Sciences, Cyprus University of Technology, Limassol, Cyprus; Faculty of Medicine, Division of Metabolic Diseases, Department of Internal Medicine, University of Debrecen, Debrecen, Hungary; Vilnius University Faculty of Medicine and Vilnius University Hospital Santaros Klinikos, Vilnius, Lithuania; Faculty of Medicine, Cantonal Hospital Zenica, University of Zenica, Zenica, Bosnia and Herzegovina; Medical University of Sofia, Sofia, Bulgaria; Faculty of Medicine, Department of Cardiology, Menoufia University, Al Minufiyah, Egypt; Department of Internal Medicine, University Hospital Centre Zagreb, and School of Medicine, University of Zagreb, Zagreb, Croatia; GENYCO Program, Comisión Honoraria para la Salud Cardiovascular, Montevideo, Uruguay; Directorate of Research, Shifa Tameer-e-Millat University, Islamabad, Pakistan; Department of Child Health, University of Benin Teaching Hospital, Benin City, Nigeria; Clinic for Heart and Circulatory Diseases, German Heart Centre Munich, Technical University Munich, Munich, Germany; German Centre for Cardiovascular Research, Partner Site Munich Heart Alliance, Munich, Germany; Department of Coronary Heart Disease and Atherosclerosis, Republican Specialized Centre of Cardiology, Ministry of Health of Republic Uzbekistan, Tashkent, Uzbekistan; Department of Vascular Medicine, D3.330, AMC, Meibergdreef 9, Amsterdam 1105 AZ, The Netherlands; Departments of Environmental and Occupational Medicine, and Internal Medicine (Cardiology Division), National Taiwan University Hospital, Taipei, Taiwan; Admiralty Medical Centre and Khoo Teck Puat Hospital, Yishun Health, Singapore; Federal State Budgetary Educational Institution of Further Professional Education “Russian Medical Academy of Continuous Professional Education” of the Ministry of Healthcare of the Russian Federation, Moscow, Russia; Lipid Clinic, Mater Dei Hospital, Msida, Malta; Faculty of Medicine and Surgery, Department of Medicine, University of Malta, Msida, Malta; Faculty of Medicine, Macau University of Science and Technology, Macau, China; Faculty of Medicine, Phenikaa University, Vietnam Atherosclerosis Society, Hanoi, Vietnam; Atherothrombosis Research Centre, University of Ioannina, Ioannina, Greece; Rigshospitalet, Copenhagen University Hospital, University of Copenhagen, Copenhagen, Denmark; Departamento Académico Ciclo de Vida, Universidad Autónoma de Guadalajara, Av. Patria 1201, Zapopan 45129, México; North Estonia Medical Centre, Tallinn University of Technology, Tallinn, Estonia; Department of Diabetology, LF SZU, Institute of Nutrition, FOaZOS, Coordination Centre for Familial Hyperlipidemias, Slovak Medical University in Bratislava, Bratislava, Slovakia; Rinku General Medical Centre, Osaka, Japan; Department of Primary Care and Public Health, Imperial Centre for Cardiovascular Disease Prevention, School of Public Health, Imperial College London, White City Campus, 90 Wood Lane, London W12 0BZ, UK

**Keywords:** Dyslipidaemia, Adiposity, Insulin resistance, Atherosclerosis

## Abstract

**Background and Aims:**

Overweight and obesity are modifiable risk factors for atherosclerotic cardiovascular disease (ASCVD) in the general population, but their prevalence in individuals with heterozygous familial hypercholesterolaemia (HeFH) and whether they confer additional risk of ASCVD independent of LDL cholesterol (LDL-C) remains unclear.

**Methods:**

Cross-sectional analysis was conducted in 35 540 patients with HeFH across 50 countries, in the EAS FH Studies Collaboration registry. Prevalence of World Health Organization–defined body mass index categories was investigated in adults (*n* = 29 265) and children/adolescents (*n* = 6275); and their association with prevalent ASCVD.

**Results:**

Globally, 52% of adults and 27% of children with HeFH were overweight or obese, with the highest prevalence noted in Northern Africa/Western Asia. A higher overweight/obesity prevalence was found in non-high-income vs. high-income countries. Median age at familial hypercholesterolaemia diagnosis in adults with obesity was 9 years older than in normal weight adults. Obesity was associated with a more atherogenic lipid profile independent of lipid-lowering medication. Prevalence of coronary artery disease increased progressively across body mass index categories in both children and adults. Compared with normal weight, obesity was associated with higher odds of coronary artery disease in children (odds ratio 9.28, 95% confidence interval 1.77–48.77, adjusted for age, sex, lipids, and lipid-lowering medication) and coronary artery disease and stroke in adults (odds ratio 2.35, 95% confidence interval 2.10–2.63 and odds ratio 1.65, 95% confidence interval 1.27–2.14, respectively), but less consistently with peripheral artery disease. Adjusting for diabetes, hypertension and smoking modestly attenuated the associations.

**Conclusions:**

Overweight and obesity are common in patients with HeFH and contribute to ASCVD risk from childhood, independent of LDL-C and lipid-lowering medication. Sustained body weight management is needed to reduce the risk of ASCVD in HeFH.

## Structured Graphical Abstract

**Structured Graphical Abstract:**
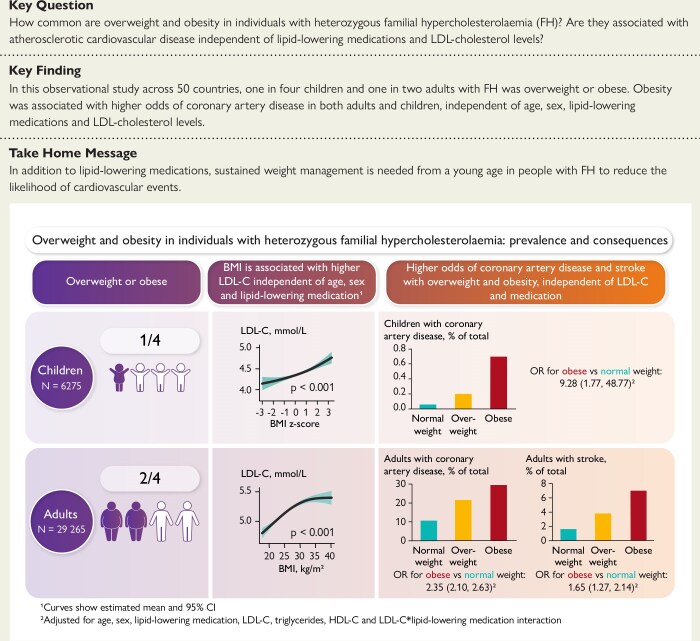
Overweight and obesity are common in patients with heterozygous familial hypercholesterolaemia and are associated with higher LDL cholesterol and greater risk of atherosclerotic cardiovascular disease, independent of lipid-lowering medication. BMI, body mass index; CI, confidence interval; HDL-C, HDL cholesterol; LDL-C, LDL-cholesterol; OR, odds ratio.

**See the editorial comment for this article ‘Obesity in familial hypercholesterolaemia: when precision medicine should meet precision population health’, by J.-P. Després, https://doi.org/10.1093/eurheartj/ehae810.**

## Introduction

Globally, cardiovascular disease (CVD) affects ∼9% of individuals and is responsible for one-third of all deaths and substantial disability.^1^ Trends over the last two decades suggest that increased body mass index (BMI)^2,3^ is a key contributor to increasing atherosclerotic CVD (ASCVD) risk worldwide.^4–6^ Atherosclerosis results from the accumulation of apoB-containing lipoproteins (mostly LDL particles)^7^ within the vessel wall, which may be accelerated by the presence of additional risk factors. Atherosclerosis development, even in the general population, often starts from childhood.^8,9^ Familial hypercholesterolaemia (FH) is increasingly recognized as a public health concern^10–12^ with an estimated worldwide prevalence of around 1:300 individuals,^13,14^ and with affected people having increased risk of premature ASCVD. The main driver of this process is the extreme elevation in LDL cholesterol (LDL-C) due to pathogenic variants in the LDL receptor (*LDLR*), apolipoprotein B (*APOB*), or proprotein convertase subtilisin/kexin type 9 (*PCSK9*) genes that impair the receptor-mediated clearance of LDL by the liver. In addition to the lifelong exposure to high LDL-C if untreated or undertreated, people with FH may be exposed to other risk factors known to further increase ASCVD risk,^15,16^ but these have been less thoroughly investigated.

Obesity is a key modifiable cardiovascular risk factor that warrants investigation in people with FH. High BMI was identified in a meta-analysis of three individual studies as one of the risk factors significantly associated with CVD in heterozygous FH (HeFH).^17^ However, data were inconsistent, with ‘obesity’ not reaching statistical significance in relation to ASCVD risk.^17^ In the SAFEHEART registry prospective analysis of 2404 patients with HeFH, BMI was associated with increased risk of a combined CVD outcome variable that encompassed myocardial infarction, cardiovascular procedures, stroke, and CVD mortality.^18^ While this provided evidence that obesity predicts adverse outcomes in FH, the contribution of obesity to individual conditions, such as coronary artery disease (CAD) or stroke was not dissected. Further, in most world regions, the prevalence of overweight and obesity in people with FH is not known—the size of the population affected with this risk factor may determine the priority of targeting it to reduce ASCVD risk. Specifically, in the present study, we assessed the prevalence of overweight and obesity in patients with HeFH in different world regions. We then investigated whether there is a graded relationship between body weight categories and ASCVD independent of the major risk drivers in FH, namely LDL-C exposure and lipid-lowering medication (LLM), and if present, how early in life does this appear. Using the FH Studies Collaboration (FHSC)^10,11,19^ registry, we attempted to resolve these uncertainties using the largest evidence base to date.

## Methods

### FHSC registry

The methods of the FHSC project have been described in detail elsewhere.^10,19^ Briefly, the FHSC registry comprises individual-level data supplied by an international consortium of investigators with access to data from patients managed in specialist clinics that serve as national, regional, or local registries of FH. The data from these diverse sources are standardized to a common data dictionary and merged into a single global registry.^19^ The structure of the FHSC Coordinating Centre, Steering, and Executive Committees has also been published previously.

The registry includes adults and children with a clinical and/or genetic diagnosis of HeFH or homozygous FH (HoFH). A clinical diagnosis follows established criteria (or modified criteria thereof), such as the Dutch Lipid Clinic Network (DLCN) criteria, Make Early Diagnoses to Prevent Early Deaths (MEDPED), Simon Broome, Canadian, or Japanese Atherosclerosis Society criteria. The FHSC registry currently consists of >72 000 patients from 73 countries, including 11 953 children and adolescents (aged <18 years).

The protocol and data governance of the registry and its use for research have been approved by the Joint Research Compliance Office and Imperial College Research Ethics Committee (Imperial College London, London, UK). Investigators contributing to the registry provide written confirmation that they comply with their local research and ethical policies and regulations for sharing data with the registry. The FHSC project is registered at ClinicalTrials.gov (NCT04272697).

### Present analysis

We conducted a cross-sectional study of adults and children with HeFH using data collected from FHSC registry inception (15 October 2015) till 20 December 2022. Individuals were included if they had a clinical and/or genetic diagnosis of HeFH and had BMI data available at registry entry. In case of adults with a clinical diagnosis, only those with a probable or definite diagnosis of FH (possible and definite in case of Simon Broome criteria) were included. In the case of children, in whom most clinical criteria do not apply, a diagnosis of FH by the treating physician was accepted, and 83% of children had a genetic diagnosis. Patients diagnosed with HoFH were excluded; additionally, participants with a clinical-only diagnosis of HeFH and an untreated LDL-C of 12.9 mmol/L (500 mg/dL) or higher were excluded, as these concentrations make the diagnosis of HoFH likely.

### Variables and subgroup definitions

Clinical and laboratory data were supplied by the individual investigators, as measured locally in the respective clinics and laboratories.

Age refers to age at the time of data collection, unless otherwise specified. Adults were defined as those aged 18 years or over at data collection; children and adolescents were those aged 5 to <18 years. The terms ‘children’ and ‘children and adolescents’ are used interchangeably throughout the manuscript to refer to the latter group of individuals. Due to greater susceptibility of their body weight to confounders not measured in the present study, children aged <5 years were not included in the study.

Data on BMI were classified into body weight categories defined according to World Health Organization (WHO) cut-offs.^20,21^ In adults, weight categories were: obese (BMI ≥30 kg/m^2^), overweight (BMI 25 to <30 kg/m^2^), normal weight (BMI 18.5 to <25 kg/m^2^), and underweight (BMI <18.5 kg/m^2^).^20^ Additionally, since Asian populations exhibit CVD risk at lower BMI measurements than Caucasians,^22^ a sensitivity analysis was conducted where lower cut-offs^22^ were used for overweight (BMI 23 to <25 kg/m^2^) and obesity (BMI ≥25 kg/m^2^) for all patients from Asia, while the standard cut-offs^20^ were retained for all other patients.

For children and adolescents, height, weight, age, and gender data were used to calculate a BMI *z*-score for each individual using the WHO growth reference data^21^ as implemented in the R statistical package ‘anthroplus’;^23^ the *z*-scores were then used to define weight categories as follows: obese (BMI *z*-score > +2SD), overweight (BMI *z*-score > +1SD and <2SD), normal weight (BMI *z*-score >−2SD and <+1SD), and underweight (BMI *z*-score <−2SD).^21^ In cases where only BMI data were provided by the investigators, but not weight or height data, BMI category was defined manually using WHO sex-specific BMI-for-age growth tables.^24,25^

The atherogenic index, a predictor of CAD, was calculated as log [triglycerides/HDL cholesterol (HDL-C)].^26,27^ Non-HDL-C was calculated as the difference between total cholesterol and HDL-C. Triglyceride-rich lipoprotein-cholesterol (TRL-C; also known as remnant cholesterol) was calculated as total cholesterol: (LDL-C + HDL-C).^28,29^

Index case was defined as the first documented FH case in a family; non-index cases were defined as relatives with FH identified through screening of the family from the index case.

Atherosclerotic CVD comprised (premature) CAD, peripheral arterial disease (PAD), and stroke. Premature CAD was defined as a CAD diagnosis before the age of 55 years in men and 65 years in women. The countries contributing data on the different types of ASCVD are listed in Supplementary data online, *Table S1*.

Hypertension was investigator-reported (‘no/yes’). Diabetes was coded as no/yes where ‘yes’ indicated Type 1 diabetes, Type 2 diabetes, or diabetes with type not specified by the investigator. Smoking was coded as ‘ever smoker’ vs. ‘never smoker’.

Geographical regions were defined according to the United Nations (UN) classification.^30^ Sub-regions were combined to increase statistical power if they included a small number of subjects and were homogeneous with respect to the prevalence of overweight and obesity in the general population and in the current data set. High-income vs. non-high-income countries were defined according to the 2024 World Bank classification of country-income status (defined according to Gross National Income per capita in 2022).^31^ The list of countries included in each UN region or income category is shown in Supplementary data online, *Tables S2–S4*.

### Statistical analysis

All analyses were conducted separately in adults and children/adolescents.

Summary data are presented as median (25th and 75th percentiles) for continuous variables. Categorical variables are reported as absolute numbers (relative frequencies from the total number of participants with data available for the corresponding variable). Group comparisons in *Table 1* were performed using Kruskal–Wallis test for continuous variables and Pearson’s χ^2^ test for categorical variables. To visualize the independent and potential non-linear relationships between BMI and plasma lipids, generalized additive linear models were fitted and plotted, separately for adults and children, with BMI or BMI *z*-score, respectively, as the independent variable, and the different lipid fractions as outcomes. Smooth terms were modelled using penalized thin-plate regression splines, with knots selected automatically and smoothing parameters optimized via generalized cross-validation as implemented in the R package mgcv.^32^ These models were adjusted for age, sex, and use of LLM. Skewed lipid variables (HDL-C, total cholesterol, and triglycerides) were log transformed for this analysis.

**Table 1:** Characteristics of the study population by age group and body weight status^a^

	Children and adolescents					Adults				
Variable	*n*	Normal weight *n* = 4608^b^	Overweight *n* = 1118	Obese *n* = 549	*P*-value	*n*	Normal weight *n* = 14 045^c^	Overweight *n* = 10 404	Obese *n* = 4796	*P*-value
Age, years	6275	11.0 (8.0, 14.4)^b^	10.8 (8.0, 13.9)	10.0 (7.9, 12.9)	*<*.*001*	29 245	42 (30, 55)	50 (39, 61)	52 (41, 61)	*<*.*001*
Sex, *n* (%)	6275				*<*.*001*	29 237				*<*.*001*
Male		2225 (48%)	564 (50%)	323 (59%)			5689 (41%)	5724 (55%)	2083 (43%)	
Female		2383 (52%)	554 (50%)	226 (41%)			8351 (59%)	4678 (45%)	2712 (57%)	
Age at FH diagnosis, years	6205	10.8 (7.8, 14.2)	10.2 (7.7, 13.0)	10.0 (7.4, 12.0)	*<*.*001*	26 104	40 (28, 53)	48 (37, 59)	49 (38, 58)	*<*.*001*
Index case, *n* (%)	5976	990 (22%)	358 (34%)	243 (48%)	*<*.*001*	21 900	2317 (21%)	2390 (31%)	1286 (40%)	*<*.*001*
Lipid-lowering medication, *n* (%)	6170	1463 (32%)	336 (31%)	134 (25%)	.*004*	28 213	8382 (62%)	7245 (72%)	3490 (76%)	*<*.*001*
Statins, *n* (%^d^)		304 (21%)	108 (32%)	53 (40%)	*<*.*001*		6489 (77%)	6254 (86%)	3109 (89%)	*<*.*001*
Smoking, *n* (%)	4849	175 (4.7%)	52 (6.5%)	20 (5.9%)	.*079*	27 609	5015 (37.8%)	3908 (39.8%)	1827 (40.5%)	*<*.*001*
Hypertension, *n* (%)	5189	5 (0.1%)	4 (0.5%)	7 (1.9%)	*<*.*001*	26 858	1584 (12%)	2534 (27%)	1826 (41%)	*<*.*001*
Diabetes, *n* (%)	4988	11 (0.3%)	3 (0.4%)	2 (0.6%)	.*40*	26 744	365 (2.8%)	606 (6.4%)	679 (15%)	*<*.*001*
Premature coronary artery disease, *n* (%)						25 083	808 (6.5%)	1251 (14%)	808 (21%)	*<*.*001*
Coronary artery disease, *n* (%)	6172	3 (<0.1%)	2 (0.2%)	4 (0.7%)	.*003*	25 720	1381 (11%)	2054 (22%)	1262 (30%)	*<*.*001*
Stroke, *n* (%)	4780	0 (0%)	0 (0%)	1 (0.3%)	.*063*	23 522	186 (1.6%)	233 (2.8%)	134 (3.7%)	*<*.*001*
Peripheral artery disease, *n* (%)	823	0 (0%)	0 (0%)	0 (0%)		8836	133 (3.8%)	181 (5.4%)	144 (7.4%)	*<*.*001*
United Nations Region	6275				<.001	29 245				*<*.*001*
Africa		0 (0.0%)	0 (0.0%)	0 (0.0%)			7 (0.0%)	25 (0.2%)	25 (0.5%)	
Americas		186 (4.0%)	56 (5.0%)	50 (9.1%)			996 (7.1%)	857 (8.2%)	457 (9.5%)	
Asia		36 (0.8%)	12 (1.1%)	5 (0.9%)			1050 (7.5%)	962 (9.2%)	580 (12.1%)	
Europe		4353 (94.5%)	1038 (92.8%)	487 (88.7%)			11 914 (84.8%)	8484 (81.5%)	3673 (76.5%)	
Oceania		33 (0.7%)	12 (1.10%)	7 (1.3%)			84 (0.6%)	88 (0.8%)	64 (1.3%)	

^a^Study population with age data available. Data are median (25th, 75th percentiles) or *n* (% of total population, unless otherwise indicated). *P*-values, in italics, are from Kruskal–Wallis test for continuous variables or Pearson’s χ^2^ test for categorical variables.

^b^ *n* = 222 underweight children/adolescents.

^c^ *n* = 574 underweight adults.

^d^Percent of those on lipid-lowering medication.

To assess the heterogeneity in lipid measurements between clusters (countries), we calculated intra-class correlation coefficients (ICCs) using a 2-way random-effects model adjusted for age, sex, and LLM. This quantified the proportion of total variance attributable to differences between countries. Intra-class correlation coefficients were low for lipids not affected by FH, i.e. triglycerides (0.05) and HDL-C (0.07), indicating minimal heterogeneity due to sampling and assay methods across countries. In contrast, higher ICCs were observed for LDL-C (0.28) and total cholesterol (0.27), likely reflecting true differences in the severity of FH-causing genetic variants across countries and regions.

The associations between BMI category and odds of CAD, premature CAD, PAD, or stroke were tested using multivariate logistic regression, using three models, in a complete case analysis. Model 1 was adjusted for age and sex; Model 2 was additionally adjusted for LLM, LDL-C, HDL-C, and triglycerides, and the interaction between LLM and LDL-C; this interaction term was included to account for the LLM effect on LDL-C and capture any effect modification caused by inter-individual variations in the LDL-C response to LLM. Model 3 included all Model 2 covariates, in addition to disease and lifestyle risk factors associated with ASCVD, namely diabetes, hypertension, and smoking. Model 2 was considered the main model because it had the lowest combined risk of over-adjustment and residual confounding. The possibilities of effect modification by age, sex, and index status were tested in this model.

Sensitivity analyses were conducted in the subgroup with a genetic diagnosis of FH. This subgroup comprised 76% (*n* = 22 337) of the total adult cohort investigated (*n* = 29 265), and 83% (*n* = 5215) of the children and adolescent cohort (*n* = 6275). Another sensitivity analysis was conducted in adults using lower overweight and obesity cut-offs for patients from Asia, as explained above.

IBM SPSS (Version 28.0; IBM Corp., Armonk, NY, USA) and R software (version 4.2.1 for Windows; R Foundation for Statistical Computing, Vienna, Austria) were used to analyse the data. All statistical tests are two-tailed, and a *P*-value <.05 was considered statistically significant.

## Results

### Prevalence of overweight and obesity

**Figure 1:**
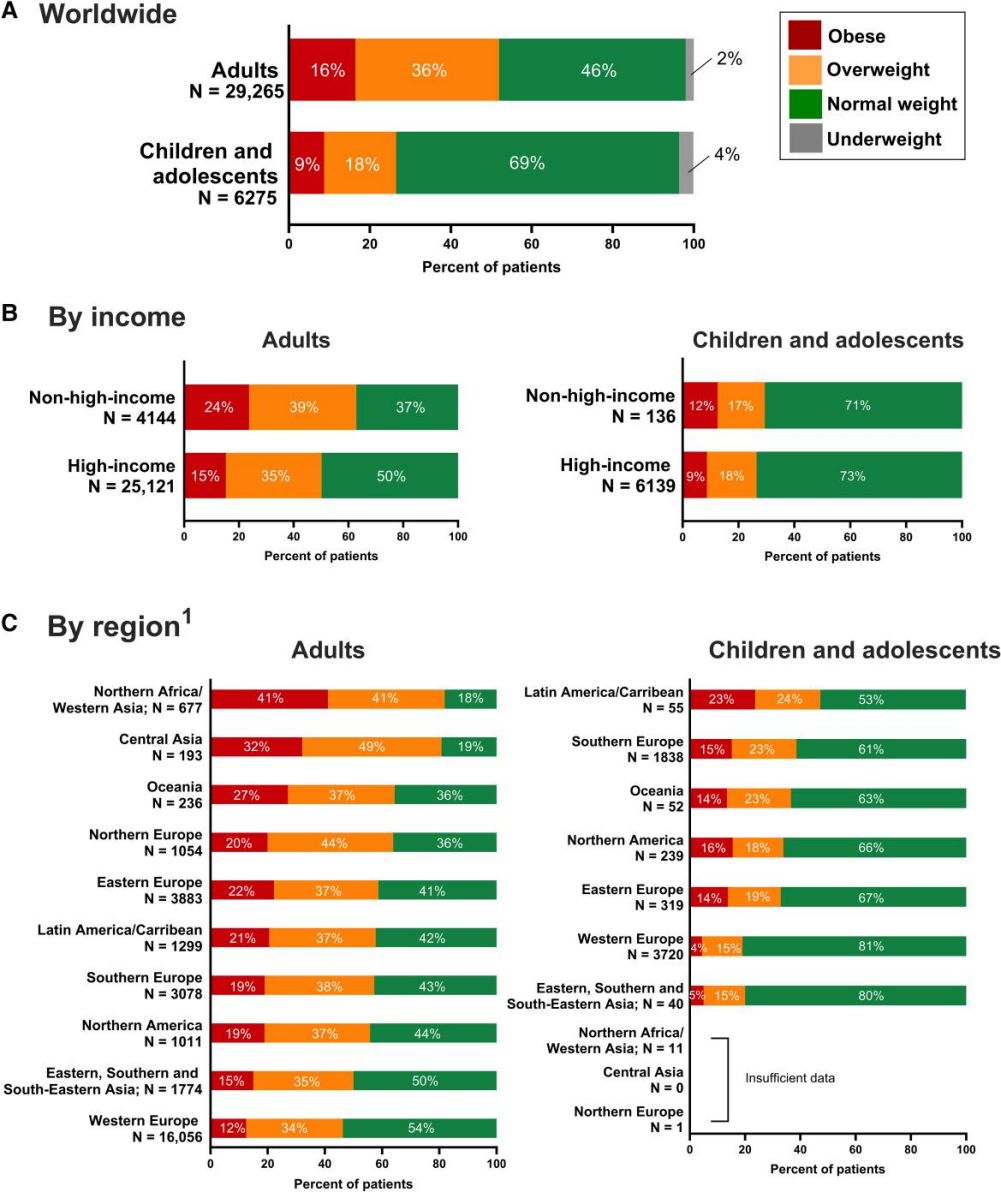
Prevalence of underweight, normal weight, overweight, and obesity in people with heterozygous familial hypercholesterolaemia pooled globally (*A*), by country-income category (*B*), and by United Nations sub-regions (*C*). Adults were aged 18 years or older; children and adolescents were aged 5 to <18 years. Body mass index categories were defined by World Health Organization body mass index cut-offs (see Methods section for details). In *B* and *C*, underweight individuals are pooled with the normal weight category; data are sorted in descending order of combined overweight and obesity prevalence. No formal statistical comparisons are conducted as these prevalence are for descriptive purposes only. ^1^*n* = 5 adults from Nigeria were not included in this analysis

The study population included 29 265 adults and 6275 children with HeFH and data available on BMI at registry entry. The BMI distribution of the study population, overall and stratified by region and country-income category, is shown in *Figure 1*. Overall, 36% of adults were overweight, and a further 16% were obese. In children and adolescents, 18% were overweight and 9% were obese. In the genetically confirmed subgroup, the prevalence were as follows: adults: 34% overweight and 14% obese; children: 17% overweight and 7% obese.

In sex-stratified analysis, more men (5727/13 508; 42%) than women (4678/15 750; 30%) were overweight, whereas obesity was more prevalent in women (17%) than men (15%). In children, both overweight and obesity were slightly more prevalent in boys than in girls (see Supplementary data online, *Table S5*).

Underweight individuals constituted only 2% of children and 4% of adults (*Figure 1A*). There was no significant difference between these individuals and normal weight individuals in the odds of any type of CVD after adjusting for age and sex (see Supplementary data online, *Table S6*). Therefore, underweight children and adults were combined with their normal weight counterparts for all subsequent analysis.

There was a higher proportion of adults with overweight or obesity (63%) in non-high-income countries compared with high-income countries (50%; *Figure 1B*).

The prevalence of overweight and obesity across the five main UN regions is shown in Supplementary data online, *Figure S1*. The prevalence of overweight and obesity was lower in Europe than any other world region; data from Africa was limited (adults, *n* = 57) or absent (children). By UN sub-region, the highest prevalence of adults classified as overweight or obese was in the Northern Africa and Western Asia region (82% with overweight or obesity), and the lowest was in Western Europe (*Figure 1C*). Among children the lowest proportion considered to be overweight or obese (approximately one in five children) was also found in Western Europe (*Figure 1C*).

### Population characteristics by weight status

Subject characteristics by BMI category are summarized in *Table 1*. Adults with HeFH who were classifiable as obese were diagnosed with HeFH 9 years later than those with normal weight (median age at FH diagnosis 49 vs. 40 years; *P* < .001). The late FH diagnosis in adults with obesity did not appear to be driven by systematic differences in the prevalence of obesity and FH detection methods across countries, since it was apparent in most UN sub-regions in stratified analysis (see Supplementary data online, *Table S7*). Conversely, in children, age at FH diagnosis was nearly a year younger in those with obesity vs. normal weight (*Table 1*). In the genetically confirmed group, similar findings were observed: age at FH diagnosis in adults in the normal weight, overweight, and obese categories, respectively, was [median (25th, 75th percentiles)] 39.0 (27.7, 52.3), 47.2 (36.0, 59.5), and 48.7 (37.5, 59.2) years. Equivalent values for genetically diagnosed children were: 11.1 (8.0, 14.5), 10.6 (8.0, 13.6), and 9.9 (7.2, 12.9) years.

In both children and adults, the proportion of patients who were index cases of HeFH increased across BMI categories; for example, 22% of normal weight children were index cases, compared with 48% of children with obesity (*P* < .001). Hypertension, diabetes, and CVD were more frequent in overweight than normal weight individuals, and even more frequent in individuals with obesity, with the exception of diabetes in children (*Table 1*).

Approximately two-thirds of children and one-third of adults were not receiving LLM at registry entry. Overall, LDL-C, non-HDL-C, TRL-C, triglycerides, and the atherogenic index were higher across higher BMI categories in both children and adults, and HDL-C was lower, irrespective of use of LLM (*Table 2*). The differences in total cholesterol and LDL-C across weight categories were generally less marked among patients who were receiving LLM than those who were untreated, and, among children receiving LLM, there was no significant difference in total cholesterol or LDL-C or by weight category classes (*Table 2*).

**Table 2:** Plasma lipid concentrations across body mass index categories by age group and lipid-lowering medication status^a^

Children and adolescents	Not receiving lipid-lowering medication					Receiving lipid-lowering medication				
	*n*	Normal weight	Overweight	Obese	*P*-value	*n*	Normal weight	Overweight	Obese	*P*-value
		*n* = 3080	*n* = 759	*n* = 398			*n* = 1463	*n* = 336	*n* = 134	
Total cholesterol, mmol/L	3428	6.30 (5.40, 7.27)	6.50 (5.62, 7.50)	6.70 (5.84, 7.80)	*<*.*001*	1818	5.82 (4.97, 6.95)	6.00 (5.09, 6.86)	6.05 (5.19, 7.10)	.*34*
LDL cholesterol, mmol/L	3297	4.57 (3.70, 5.51)	4.76 (3.92, 5.74)	4.90 (3.99, 6.09)	*<*.*001*	1792	4.19 (3.36, 5.20)	4.38 (3.46, 5.17)	4.39 (3.53, 5.35)	.*51*
HDL cholesterol, mmol/L	3398	1.30 (1.06, 1.52)	1.27 (1.07, 1.50)	1.27 (1.03, 1.50)	.*22*	1806	1.21 (1.01, 1.42)	1.14 (0.96, 1.40)	1.12 (0.91, 1.32)	*<*.*001*
Triglycerides, mmol/L	2507	2.02 (1.40, 2.82)	2.22 (1.63, 3.32)	2.52 (1.83, 3.70)	*<*.*001*	1760	1.91 (1.37, 2.68)	2.07 (1.53, 2.90)	2.51 (1.68, 3.71)	*<*.*001*
TRL cholesterol, mmol/L	3180	0.40 (0.30, 0.57)	0.44 (0.31, 0.64)	0.49 (0.34, 0.78)	*<*.*001*	1761	0.39 (0.27, 0.54)	0.41 (0.30, 0.60)	0.50 (0.32, 0.76)	*<*.*001*
Non-HDL cholesterol, mmol/L	959	5.30 (4.50, 6.34)	5.61 (4.64, 6.70)	5.90 (4.97, 6.87)	*<*.*001*	383	4.97 (3.83, 6.10)	5.00 (3.84, 5.89)	4.81 (4.25, 6.40)	.*8*
Atherogenic index	2492	0.48 (0.08, 0.93)	0.62 (0.26, 1.10)	0.74 (0.32, 1.25)	*<*.*001*	1757	0.46 (0.09, 0.90)	0.56 (0.22, 0.99)	0.83 (0.37, 1.22)	*<*.*001*

Adults	*n*	*n* = 5246	*n* = 2764	*n* = 1086	*P*-value	*n*	*n* = 8382	*n* = 7245	*n* = 3490	*P-value*
Total cholesterol, mmol/L	5902	7.11 (5.93, 8.46)	7.70 (6.50, 9.10)	7.89 (6.65, 9.20)	*<*.*001*	16 720	5.95 (5.00, 7.49)	6.04 (4.99, 7.73)	6.26 (5.08, 8.03)	*<*.*001*
LDL cholesterol, mmol/L	5779	5.16 (4.10, 6.41)	5.60 (4.63, 6.85)	5.76 (4.69, 6.98)	*<*.*001*	16 423	4.05 (3.11, 5.38)	4.09 (3.10, 5.48)	4.29 (3.19, 5.87)	*<*.*001*
HDL cholesterol, mmol/L	5723	1.32 (1.07, 1.61)	1.22 (0.99, 1.50)	1.19 (0.99, 1.47)	*<*.*001*	16 280	1.33 (1.09, 1.60)	1.20 (0.98, 1.47)	1.14 (0.95, 1.38)	*<*.*001*
Triglycerides, mmol/L	4761	2.51 (1.73, 3.64)	3.44 (2.41, 4.91)	4.12 (2.83, 5.94)	*<*.*001*	15 392	2.31 (1.65, 3.39)	3.10 (2.18, 4.54)	3.71 (2.56, 5.25)	*<*.*001*
TRL cholesterol, mmol/L	5573	0.50 (0.34, 0.73)	0.69 (0.48, 0.98)	0.80 (0.54, 1.16)	*<*.*001*	15 775	0.47 (0.32, 0.70)	0.60 (0.41, 0.91)	0.70 (0.49, 1.01)	*<*.*001*
Non-HDL cholesterol, mmol/L	5713	5.71 (4.57, 6.98)	6.37 (5.26, 7.70)	6.62 (5.38, 7.88)	*<*.*001*	16 251	4.55 (3.58, 6.05)	4.71 (3.66, 6.28)	4.94 (3.81, 6.68)	*<*.*001*
Atherogenic index	4707	0.63 (0.19, 1.10)	1.05 (0.57, 1.51)	1.25 (0.74, 1.69)	*<*.*001*	15 253	0.55 (0.14, 1.02)	0.94 (0.49, 1.43)	1.17 (0.70, 1.64)	*<*.*001*

^a^Data are median (25th, 75th percentiles). *P*-values, in italics, are from Kruskal–Wallis test.

### Association between body mass index and lipid levels

**Figure 2:**
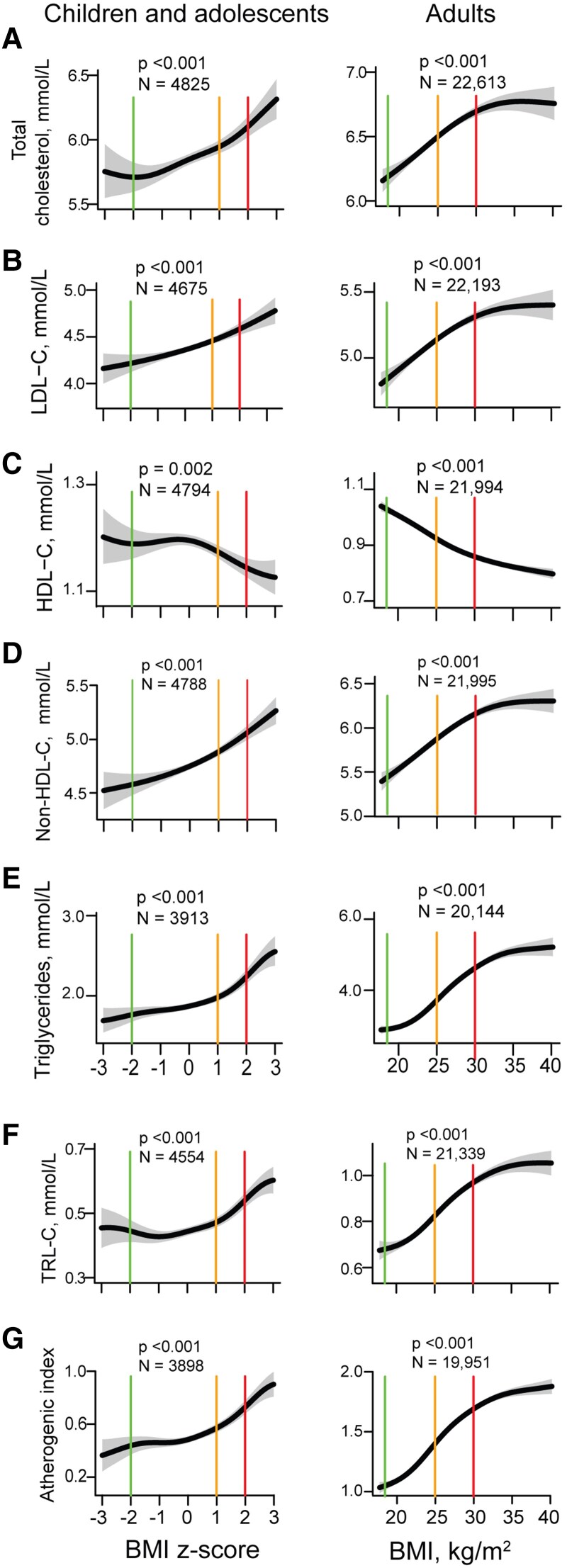
A-G: Estimated means and 95% confidence intervals of different lipid fractions by body mass index *z*-score (in children and adolescents aged 5 to <18 years; left panels) and body mass index in adults (aged ≥18 years; right panels) with adjustment for age, sex, and use of lipid-lowering medication. The vertical lines (green, yellow, and red) mark the beginning of the normal weight, overweight, and obese BMI ranges, respectively. The lowest and highest 1% of the independent variable are not shown. Note the different scales on the *y*-axes for children and adults. BMI, body mass index; TRL-C, triglyceride-rich lipoprotein cholesterol

In generalized additive linear models adjusted for age, sex, and LLM, BMI in adults and BMI *z*-score in children were positively associated with total cholesterol, LDL-C, non-HDL-C, and triglycerides (all *P* < .001) and inversely associated with HDL-C (*Figure 2*). In adults, the plots showed stronger associations across the normal weight range, with attenuation of the association in the obese BMI range. In contrast, among children, there was no attenuation of the association at higher BMI.

An increase in BMI from the start of the normal weight range (18.5 kg/m^2^) to the BMI cut-off for obesity (30 kg/m^2^) was associated with an ∼0.5 mmol/L higher LDL-C, 1.8 mmol/L higher triglycerides, and 0.2 mmol/L lower HDL-C in adults, after controlling for age, sex, and LLM. The equivalent differences in children were: LDL-C, 0.3 mmol/L; triglycerides, 0.6 mmol/L; and HDL-C, 0.07 mmol/L, respectively. A higher BMI in adults and children was also positively associated with TRL-C and the atherogenic index (both *P* < .001; *Figure 2*). Essentially similar associations to those in the total cohort were observed in the cohort with genetically confirmed HeFH (all *P* < .001; Supplementary data online, *Figure S2*).

### Associations of overweight and obesity with atherosclerotic cardiovascular disease

Coronary artery disease was present in 0.7% of children with obesity, 0.2% of children with overweight, and 0.07% of normal weight children (total *n* with CAD = 9; *P* < .001; *Table 1*). Obesity was associated with significantly higher odds of CAD in children, but the confidence intervals (CIs) were wide due to the small number of events. The odds ratio (OR) in children with obesity vs. normal weight, adjusted for age and sex (Model 1) was 12.29 (95% CI: 2.96–51.03; *P* < .001). After adjustment for lipids and use of LLM (Model 2), the OR was 9.28 (95% CI 1.77–48.77; *P* = .008). None of the children had PAD at registry entry. One child had a history of stroke and a BMI in the obese range (*Table 1*).

**Figure 3:**
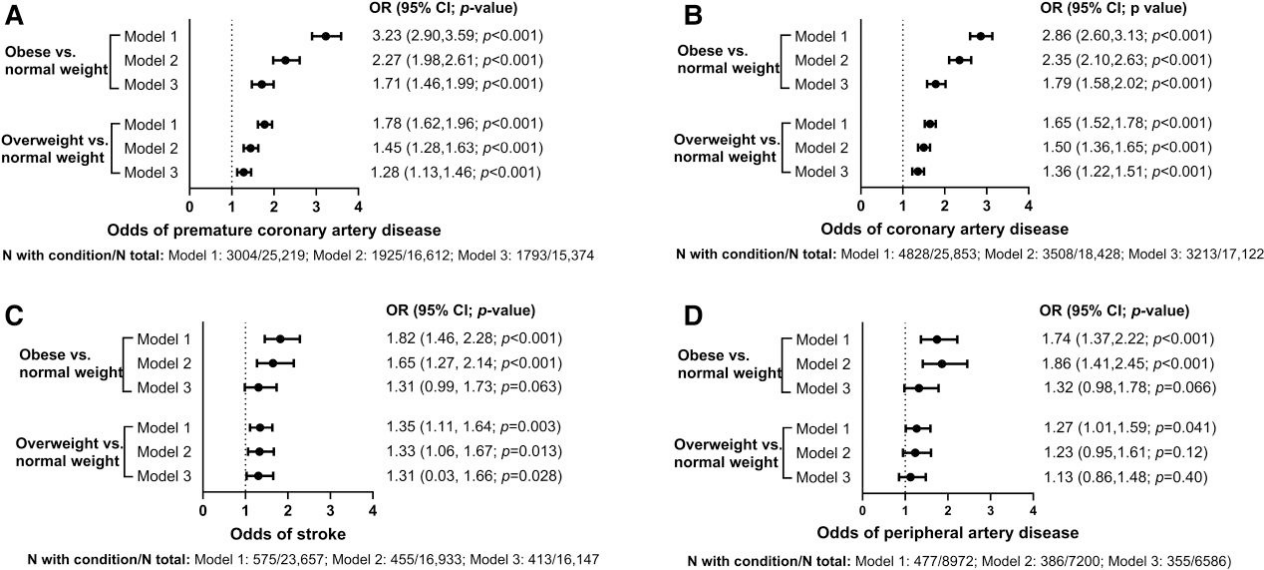
A-D: Odds ratios and 95% confidence intervals for the presence of different types of cardiovascular disease in adults with heterozygous familial hypercholesterolaemia at registry entry by body mass index category. Models are adjusted for the following variables: Model 1, age and sex; Model 2, age, sex, lipid-lowering medication, LDL cholesterol, triglycerides, HDL cholesterol, and LDL cholesterol × lipid-lowering medication interaction; Model 3, all Model 2 variables, plus diabetes, hypertension, and smoking. Body mass index categories were defined by World Health Organization body mass index cut-offs (see Methods section for details)

Adults with obesity had three times higher prevalence of CAD than normal weight adults (30% vs. 11%; *Table 1*). The prevalence of stroke and PAD in adults was low overall, but it was twice as high among those with obesity (3.7% and 7.4%, respectively) than in those with normal weight (1.6% and 3.8%). Being overweight or obese, vs. having normal weight, was associated with ∼1.5-fold and 2.3-fold higher odds of having CAD or premature CAD (all, *P* < .001; Model 2; *Figure 3A* and *B*). Further adjustment for age at FH diagnosis or index case status did not alter the results (data not shown). Odds ratios for stroke and PAD were also higher among adults with overweight and obesity vs. normal weight, but the magnitude of the associations was lower than that for CAD (*Figure 3C* and *D*). With further adjustment for diabetes, hypertension, and smoking (Model 3), all associations were attenuated, but were still statistically significant for CAD and premature CAD, borderline significant for stroke, and no longer significant for PAD (*Figure 3*). In sensitivity analysis using lower overweight and obesity cut-offs for patients from Asian countries, findings were similar to or stronger than in the primary analysis (see Supplementary data online, *Table S8*).

In the subgroup with genetically confirmed FH, essentially similar significant associations of overweight and obesity with CAD, premature CAD, and stroke were observed as those in the total population, but those with PAD were weaker and not statistically significant (see Supplementary data online, *Table S9*).

Supplementary data online, *Table S10* shows the odds of ASCVD by BMI category in different adult subgroups in the total population. No evidence of effect modification by sex was noted for any ASCVD outcome, but the association of BMI category with CAD appeared stronger in younger vs. older adults (*P*-interaction < .001), and that of BMI category with PAD tended to be stronger in non-index vs. index cases (*P*-interaction = .060).

## Discussion

Among 35 540 individuals with HeFH, approximately half of adults and a quarter of children were found to be overweight or obese. This was more marked in regions of the world where in the general population obesity is more prevalent than other regions. Among both adults and children with HeFH, overweight and obesity were associated with a more atherogenic lipid profile and with higher odds of ASCVD, independent of age, sex, lipid profile, and use of LLM (*Structured Graphical Abstract*). In adults, obesity was associated with FH being diagnosed almost a decade later than in those with normal weight. Given the late age of diagnosis of FH globally (average 44 years),^10^ and the higher prevalence of FH in some regions of the world where obesity is also common, the present findings highlight the importance of a global risk factor management approach including addressing lifestyle factors in those with FH, as recommended in most guidelines.^33^

The high prevalence of overweight and obesity found in the present study should be viewed in the context of the population prevalence of these conditions worldwide.^34^ Globally, obesity prevalence in the general adult population in 2016 was estimated at 13% of men and 15% of women^34^; in children, the prevalence was 5.6% of girls and 7.8% of boys.^34^ Bearing in mind the methodological differences across studies, the prevalence of obesity among adults (16%) and children (9%) appears similar to the general population. In the general population, compared with normal weight adults, people with overweight and obesity, respectively, have a 20% and 60% higher risk of CAD.^4^ Stroke and PAD in the general population are also linked to weight in a graded fashion, with those with obesity having the highest risk.^5^ In patients with HeFH, we observed two times higher odds of CAD and 60%–80% higher odds of stroke and PAD in adults with obesity compared with normal weight adults, independent of age, sex, lipids, and LLM. In adults, the increase of LDL-C with greater BMI was in fact attenuated at the highest BMI; this is in line with findings in the general population,^35^ possibly resulting from reduced intestinal cholesterol absorption in severe obesity,^36^ which may partly explain why the obesity–ASCVD association was independent of LDL-C.

We tested whether diabetes, hypertension, and smoking explained some of the associations of obesity with ASCVD. Adjusting for these factors weakened the associations, but a substantial independent contribution of obesity remained for CAD and stroke. This is consistent with findings in 1.8 million people from the general population that treating hypertension, diabetes, and hyperlipidaemia only mitigates half the excess risk of CAD and three quarters of the excess risk of stroke associated with high BMI.^37^ The residual risk might be explained by other factors not evaluated in the present study, including endothelial dysfunction, thrombogenesis, and inflammation.^38^ While infiltration of apolipoprotein B–containing lipoproteins, most notably LDL-C, in the artery wall is a critical event initiating atherosclerosis, it is the subsequent recruitment of monocytes and development of foam cells, inflammation and endothelial activation, that culminate in forming an atherosclerotic plaque in the vessel wall.^39^ If the fibrous cap of the plaque ruptures, it facilitates thrombogenesis, leading to ischaemic events. At all these pathophysiologic stages, obesity and the associated insulin resistance contribute to phenotypes that accelerate atherothrombosis, including increased apolipoprotein B–containing lipids, systemic inflammation, and hypercoagulability.^40^ Crucially, in the general population, intentional weight loss ameliorates these processes^41^ and reduces CVD events.^42,43^ Our findings that the pattern of association of obesity with ASCVD in patients with FH parallels that in the general population suggests that patients with FH stand to equally benefit from maintaining a healthy weight with regards to ASCVD, on top of their benefit from pharmacologic LDL-C reduction.

Atherosclerosis begins in childhood, with obesity accelerating the process through insulin resistance, which may directly affect the vessel wall or contribute to atherogenic dyslipidaemia, such as lower HDL-C and higher remnant cholesterol in triglyceride-rich lipoproteins.^44^ In the general paediatric population, obesity has been linked to endothelial dysfunction and inflammation, starting as early as age 8,^45,46^ and to a higher risk of stroke before age 50,^47^ but ASCVD events in childhood are rare. In children with HeFH, where atherosclerosis is accelerated due to high LDL-C from birth, clinical ASCVD also remains uncommon. Our recent analysis of over 11 000 children and adolescents with HeFH, the largest such data set to date, revealed a CAD prevalence of 0.3% and a stroke prevalence of <0.1%.^48^ In the present study of those with BMI data, the occurrence of CAD was 10 times higher in children with obesity (0.7%) vs. those with normal weight (0.07%). Due to the small total number of events these results should be interpreted with caution, but the higher occurrence of CAD in children with obesity was consistent with the higher levels of atherogenic lipids observed in this group. Childhood, therefore, comprises a window of opportunity not only for FH detection and treatment to reduce lifetime LDL-C exposure, but also for management of traditional ASCVD risk factors, including obesity, to decrease ASCVD events.^8,49^

To our knowledge, this is the first study to systematically investigate the prevalence of obesity and its contribution to ASCVD in children and adults with HeFH globally using individual-level data, but there have been relevant reports from individual countries. In the Dutch HeFH registry cohort, higher BMI was associated with a higher OR for CAD independent of LDL-C, but it was not clear whether it was also independent of LLM intake.^50^ In another study of 2400 Dutch patients, those who developed CVD had higher mean BMI.^51^ In an early study of 120 Canadian men with FH, high waist circumference and hyperinsulinaemia markedly increased the odds of CAD, independent of LDL-C.^52^ The present study consolidates and extends these findings using clinically relevant body weight categories, showing that obesity and overweight are common in FH, and increase the likelihood of both CAD and stroke across the life-course starting in childhood, regardless of LDL-C and LLM intake.

Screening for FH is not systematically performed in most healthcare systems; hence, it is often the clinical phenotype that triggers investigations for FH. Consistent with this, people with obesity in the present study were more likely to be index cases, i.e. the first case of FH discovered in a family. Our data show an interesting distinction between adults and children which warrants discussion. Children with obesity were on average, nearly 1 year younger at FH diagnosis than normal weight children, but adults with obesity were 9 years older at FH diagnosis. These could be explained by biases in detection due to health perception. Children are not expected to be obese, and this is considered unhealthy and leads to investigations, hence, FH detection at a slightly younger age in children with obesity. In contrast, there may be a propensity in adult healthcare to attribute hyperlipidaemia and/or ASCVD to the obese state, rather than consider an FH diagnosis. Thus, obesity may confer a disadvantage later in life regarding FH detection. Additionally, obesity could delay a diagnosis of FH by making it more challenging to distinguish from phenotypically similar conditions such as mixed dyslipidaemia resulting from insulin resistance. Further, a mixed dyslipidaemia phenotype may result in some individuals being considered to have familial combined hyperlipidaemia^53^ rather than FH and obesity. In our analyses of genotypically confirmed FH, the same associations were observed.

### Strengths and limitations

The present study provides the most reliable evidence to date of the prevalence of obesity and its association with ASCVD in individuals with HeFH, drawn from 50 countries across 6 continents. This allowed some comparison of obesity prevalence across geographical regions and World Bank income groups; however, there was an over-representation of Europe and high-income countries. Additional strengths include comparison of the child and adult populations, providing insights for public health strategies from childhood. However, the data are cross-sectional, and while a large body of evidence in the general population links obesity to ASCVD causation, reverse causality cannot be excluded. Further, despite the value of BMI in categorizing overweight and obesity, its limitations in not distinguishing adiposity or body fat distribution are well recognized. It was not possible to assess the association of central obesity with ASCVD due to the absence of data on waist–hip ratio in most patients, so this warrants investigation in future studies. To account for the fact that populations of different ethnicities have different body fat% or cardio-metabolic risk at the same BMI,^22^ we analysed our data using the global WHO cut-offs for overweight/obesity, as well as using lower cut-offs for Asian patients, and the associations of weight with ASCVD were similar. Thus, although the proportion of Caucasian patients was higher than other ethnicities in the present study, we believe our findings regarding the association of obesity with ASCVD are generalizable to all patients with FH globally. On the other hand, the prevalence of overweight and obesity in world regions, sub-regions or country-income groups with a relatively small number of patients (e.g. Oceania, various Asia sub-regions, and non-high-income countries especially children’s data) should be interpreted with caution and require investigating in larger data sets. It is possible that some patients with a clinical FH diagnosis were not true FH patients; however, in sensitivity analyses using only those with a genetically confirmed diagnosis, the relationship between weight and odds of ASCVD was still present. Finally, we adjusted for potential confounders in the obesity–ASCVD associations, but there may have been residual confounding from unmeasured variables, such as diet, physical activity and socio-economic state.

## Conclusions

The data from 6275 children and 29 265 adults provide evidence that obesity is common in those with HeFH and is associated with a more severe hyperlipidaemia phenotype and greater likelihood of ASCVD from childhood. Given their already augmented risk of ASCVD, patients with FH should be prioritized from the point of FH diagnosis for intensive lifestyle management aimed at maintaining a healthy weight, to reduce their lifetime risk of CVD events. A holistic approach, integrating body weight management with LDL-C-lowering treatments, should be used to improve cardiovascular outcomes in people with FH.

## Supplementary Material

ehae791_Supplementary_Data

## Declarations

### Data Availability

The data are not available for public sharing, due to the restrictions of the FHSC Data Sharing Agreements.

### Pre-registered Clinical Trial Number

The FHSC project is registered at ClinicalTrials.gov (NCT04272697).
