# Supplementary material for: Overweight, obesity, and cardiovascular disease in heterozygous familial hypercholesterolaemia: the EAS FH Studies Collaboration registry
Source: Eur Heart J. 2025 Jan 13;46(12):1127–40. doi: 10.1093/eurheartj/ehae791 (PMC11931214; doi:10.1093/eurheartj/ehae791)
Supplement: ehae791_Supplementary_Data [file ehae791_supplementary_data.docx]

**Prevalence of overweight and obesity among children and adults with heterozygous familial hypercholesterolemia in the EAS-FH Studies-Collaboration Registry, and their associations with cardiovascular disease**

**SUPPLEMENTARY MATERIAL**

**Contents**

[Appendix List of Contributors to Country Data for Current Study 2](#_Toc183606669)

[EAS Familial Hypercholesterolaemia Studies Collaboration (FHSC) Committees and National Lead Investigators 12](#_Toc183606670)

[Supplementary Tables 15](#_Toc183606671)

[Table S1. List of countries contributing data to the analysis of ASCVD 15](#_Toc183606672)

[Table S2. List of countries included, and number of patients contributed to the present study by United Nations region 16](#_Toc183606673)

[Table S3. List of countries included, and number of patients contributed to the present study by World Bank income category 18](#_Toc183606674)

[Table S4. List of countries included and number of patients contributing to the present study by United Nations sub-region 19](#_Toc183606675)

[Table S5. Prevalence of different body weight categories by sex 21](#_Toc183606676)

[Table S6. Prevalence and odds of ASCVD in underweight vs. normal weight adults with HeFH 22](#_Toc183606677)

[Table S7. Age (years) at FH diagnosis in adults by body weight category in different United Nations sub-regions 23](#_Toc183606678)

[Table S8. The association of overweight and obesity with ASCVD in the total adult population from all world regions, using lower BMI cutoffs for the Asian population^1^ 24](#_Toc183606679)

[Table S9. Association of overweight and obesity with ASCVD in adults with genetically confirmed FH^1^ 25](#_Toc183606680)

[Table S10. Association of overweight and obesity with ASCVD in different adult subgroups^1^ 26](#_Toc183606681)

[Supplementary Figures 27](#_Toc183606682)

[Supplementary Figure S1. 27](#_Toc183606683)

[Supplementary Figure S2. 28](#_Toc183606684)

# Appendix List of Contributors to Country Data for Current Study

**ARGENTINA:** Laura Schreier (Lab. of Lipids and Atherosclerosis. Faculty of Pharmacy and Biochemistry. University of Buenos Aires). **AUSTRALIA:** Jing Pang (School of Medicine, Faculty of Health and Medical Sciences, University of Western Australia, Perth). **AUSTRIA:** Christoph Ebenbichler (Dept. of Internal Medicine I, Medical University of Innsbruck), Hans Dieplinger (Institute of Genetic Epidemiology, Medical University of Innsbruck), Reinhold Innerhofer (Department of Laboratory Medicine), Yvonne Winhofer-Stöckl and Moritz Ferch (Division of Endocrinology & Metabolism, Dept. of Medicine III, Medical University of Vienna), Susanne Greber-Platzer and Margot Baumgartner-Kaut (Dept. of Pediatrics & Adolescent Medicine, Medical University of Vienna), Konstantin Krychtiuk, Lukas Galli and Walter Speidl (Division of Cardiology, Dept. of Internal Medicine II, Medical University of Vienna), Hermann Toplak (Division of Endocrinology and Diabetology, University of Graz), Kurt Widhalm (Austrian Academic Institute for Nutritional Medicine, Vienna), Thomas Stulnig and Michaela Stögerer-Lanzenberger (Dept. of Endocrinology and Nephrologie, Klinik Hietzing, Vienna), Kurt Huber (Dept. of Cardiology, Klinik Ottakring, Vienna), Florian Höllerl (Dept. of Diabetology, Endocrinology & Nephrology, Klinik Landstrasse, Vienna), Gersina Rega-Kaun (Dept. of Endocrinology, Rheumatology & Acute Geriatrics, Klinik Ottakring, Vienna), Lucas Kleemann (Dept. of Internal Medicine I, Universitätsklinikum Krems), Martin Mäser (Dept. of Pediatrics, Academic Teaching Hospital, Landeskrankenhaus Feldkirch), Daniela Karall and Sabine Scholl-Bürgi (Dept. of Pediatrics I, University Clinic Innsbruck, Medical University of Innsbruck), Christoph Säly (Vivit Institute, Feldkirch), Florian J. Mayer (Dept. of Laboratory Medicine, Medical University of Vienna). **BELGIUM** Alexandra Sperone (Pôle hospitalier Jolimont), Chloé Tanghe (Pôle hospitalier Jolimont) Anne-Catherine Gérard (Pôle hospitalier Jolimont). **BOSNIA AND HERZEGOVINA:** Lamija Pojskic (Cantonal Hospital Zenica), Ibrahim Sisic (Cantonal Hospital Zenica), Azra Durak Nalbantic (University Clinical Center Sarajevo). **BRAZIL:** Cinthia E. Jannes (Heart Institute InCor University of Sao Paulo), Alexandre C. Pereira (Heart Institute InCor University of Sao Paulo), Jose E. Krieger (Heart Institute InCor University of Sao Paulo). **BULGARIA:** Ivo Petrov (Dept. of Angiology and Electrophysiology, City Clinic, Sofia), Assen Goudev (University Hospital Queen Joanna, Clinic of Cardiology, Sofia), Fedya Nikolov (Dept. of Cardiology, Medical University of Plovdiv, Plovdiv), Snejana Tisheva (Dept. of Cardiology, Pulmonology and Endocrinology, Medical University of Pleven), Yoto Yotov (Dept. of Cardiology, Medical University of Varna), Ivajlo Tzvetkov (Dept. of Cardiology, Saint Anna University Hospital, Sofia). **CANADA:** Alexis Baass (McGill University); Jean Bergeron (CHU de Québec-Université Laval); Sophie Bernard (Université de Montreal); Diane Brisson (Ecogene-21 and Université de Montreal); Liam R. Brunham (University of British Columbia), Lubomira Cermakova (University of British Columbia); Patrick Couture (CHU de Québec-Université Laval); Gordon A. Francis (University of British Columbia), Daniel Gaudet (Ecogene-21 and Université de Montreal); Robert A. Hegele (Western University); Iulia Iatan (University of British Columbia); Etienne Khoury (Ecogene-21 and Université de Montreal); G.B. John Mancini (University of British Columbia); Brian W. McCrindle (University of Toronto); Martine Paquette (Montreal Clinical Research Institute); Isabelle Ruel (Research Institute of the McGill University Health Centre). **CHILE:** Ada Cuevas (Clínica Las Condes). **CHINA – HONG KONG:** Elaine Chow (The Chinese University of Hong Kong). **CROATIA:** Ivan Pećin (Dept. of Internal medicine, University Hospital Centre Zagreb, School of Medicine University of Zagreb), Dražen Perica (Dept. of Internal medicine, University Hospital Centre Zagreb, School of Medicine University of Zagreb). **CYPRUS:** Phivos Symeonides (Department of Pharmacy, Frederick University, Nicosia, Cyprus), Efstratios

Trogkanis (Department of Cardiology, Limassol General Hospital, Cyprus), Andreas Kostis (Department of

Internal Medicine, Limassol General Hospital, Cyprus), Andreas Ioannou (Department of Internal Medicine,

Nicosia General Hospital, Cyprus), Angeliki Mouzarou (Department of Cardiology, Paphos General Hospital,

Cyprus), Anthoula Georgiou (Department of Internal Medicine, Paphos General Hospital, Cyprus), Andreas

Stylianou (Department of Internal Medicine, Larnaca General Hospital, Cyprus), George Miltiadous

(Hippocrateon Private Hospital Nicosia, Cyprus), Paris Iacovides (P. Iacovides Paediatric Center, Nicosia,

Cyprus), Constantinos Deltas (Center of Excellence in Biobanking and Biomedical Research, University of

Cyprus). **CZECH REPUBLIC:** National MedPed adult centres: Michal Vrablik, Richard Ceska (General University Hospital and 1st Medical Faculty, Charles University, Prague), Vladimir Soska (St. Anne’s University Hospital, Brno), Lukas Tichy (Centre of Molecular Biology and Gene Therapy, University Hospital Brno, Brno). Regional MedPed adult centres and collaborators: Vera Adamkova, Jana Franekova (Institute for Clinical and Experimental Medicine, Prague), Renata Cifkova (Thomayer Hospital and 1st Medical Faculty, Charles University, Prague), Pavel Kraml, Katerina Vonaskova (University Hospital Kralovske Vinohrady and 3rd Medical Faculty, Prague), Jana Cepova (University Hospital Motol, Prague), Magdalena Dusejovska (General University Hospital and 1st Medical Faculty, Charles University, Prague), Lenka Pavlickova (Hospital Na Homolce, Prague), Vladimir Blaha (University Hospital and Medical Faculty, Charles University, Hradec Kralove), Hana Rosolova, Barbora Nussbaumerova, Roman Cibulka (University Hospital and Medical Faculty, Charles University, Plzen), Helena Vaverkova, Lubica Cibickova (University Hospital and Medical Faculty, Palacky University, Olomouc), Zdenka Krejsova (Lipid Outpatient Clinic, Karlovy Vary), Katerina Rehouskova (Regional Hospital Ceske Budejovice), Pavel Malina (Hospital Pisek), Milena Budikova (Outpatient Interkrim Clinics, Usti nad Labem), Vaclava Palanova (Regional Hospital Liberec), Lucie Solcova (Regional Hospital Trutnov), Alena Lubasova (Hospital Jablonec nad Nisou), Helena Podzimkova (Regional Hospital Jicin), Juraj Bujdak (Hospital of the Pardubice region), Jiri Vesely (Edumed, Nachod), Marta Jordanova, Tomas Salek (Regional T. Bata Hospital, Zlin), Robin Urbanek (Ormiga Medical House, Zlin), Stanislav Zemek (Lipid Outpatient Clinics, Uherske Hradiste), Jan Lacko (Hospital Trebic), Hana Halamkova, Sona Machacova (Hospital Vyskov), Sarka Mala (Outpatient Mephacentrum Clinics, Ostrava), Eva Cubova (Ostrava City Hospital, Ostrava), Katerina Valoskova (Hospital Frydek-Mistek), Lukas Burda (Hospital Krnov). **DENMARK:** Marianne Benn (Dept. of Clinical Biochemistry, Rigshospitalet, Copenhagen; Dept. of Clinical Medicine, Faculty of Health and medical Sciences, University of Copenhagen). **EGYPT:** Ahmed Bendary (Benha University, Faculty of medicine, Cardiology Dept.), Ihab Daoud (Italian Hospital, Cairo), Sameh Emil (Military medical academy), Atef Elbahry (Port-Foad cardiac Centre), Samir Rafla (Alexandria University, Faculty of medicine, Cardiology Dept.), Osama Sanad (Benha University, Faculty of medicine, Cardiology Dept.), Ghada Kazamel (National Heart Institute, Giza), Dr Mohamed Ashraf (National Heart Institute, Giza), Mohamed Sobhy (Alexandria University, Faculty of medicine, Cardiology Dept.), Amro El-Hadidy (Cairo University, Critical care Dept.), Mohamed Abdoul Shafy (Benha University, Faculty of medicine, Cardiology Dept.), Saif Kamal (Hayat hospital, Cairo), Mohamed Bendary (Cairo University, National Cancer Institute, Biostatistics Dept.). **ESTONIA:** Grete Talviste (Tallinn University of Technology). **GERMANY**: Jutta Christmann (DACH Society for the Prevention of Heart and Circulatory Diseases registered society, Hamburg), Alexander Dressel (DACH Society for the Prevention of Heart and Circulatory Diseases registered society, Hamburg), Felix Fath (DACH Society for the Prevention of Heart and Circulatory Diseases registered society, Hamburg), Chiara Ferraro (DACH Society for the Prevention of Heart and Circulatory Diseases registered society, Hamburg), Lydia Frenzke (DACH Society for the Prevention of Heart and Circulatory Diseases registered society, Hamburg), Alica Gopon (DACH Society for the Prevention of Heart and Circulatory Diseases registered society, Hamburg), Isabel Klein (DACH Society for the Prevention of Heart and Circulatory Diseases registered society, Hamburg), Dominika Pienkowska (DACH Society for the Prevention of Heart and Circulatory Diseases registered society, Hamburg), Tobias Sietmann (DACH Society for the Prevention of Heart and Circulatory Diseases registered society, Hamburg), Antonia Sonntag (DACH Society for the Prevention of Heart and Circulatory Diseases registered society, Hamburg), Omar Adjan (private practice, Passau), Philipp Bahrmann (private practice, Brackenheim), Andrea Baessler (Lipid Clinic; Clinic and Policlinic for Internal Medicine II; University Clinic Regensburg), Rasmus Barkowski (Charité, Berlin), Raffi Beckerdjian (Robert-Bosch-Hospital, Stuttgart), Christina Berr (University Clinic Augsburg), Andreas Birkenfeld (University Clinic Dresden), Gereon Böll (Clinic Königsfeld der Deutschen Rentenversicherung Westfalen, Ennepetal), Avisha Carstensen (Robert-Bosch-Hospital, Stuttgart), Ilja Demuth (Charité, Berlin), Holger Finkernagel (private practice, Bad Berleburg), Ioanna Gouni-Berthold (University Clinic Köln), Harry Hahmann (private practice, Insy), Michael Hamerle (University Clinic Regensburg), Julian Halder (private practice, Holzgerlingen), Maria Heide (University Clinic Greifswald), Ulrich Julius (Dept. of Internal Medicine III, University Hospital Carl Gustav Carus at the Technische niversität Dresden), Ursula Kassner (Charité, Berlin), Julius L Katzmann (Clinic and Policlinic for Cardiology, Dept. for Internal Medicine, Neurology and Dermatology, University Hospital Leipzig), Anja Kirschbaum (Reha-Klinik Königsfeld, Ennepetal), Gerald Klose (Joint Practice for Internal Medicine, Gastroenterology and Cardiology Beckenbauer & Maierhof, Bremen), Stephanie Könemann (University Clinic, Greifswald), Christel König (Clinic of Internal Medicine, Lipid Clinic, Klinikum Links der Weser, Bremen), Wolfgang König (Clinic for Heart and Circulatory Diseases, German Heart Centre Munich, Technical University Munich, Munich; DZHK German Centre for Cardiovascular Research, Partner Site Munich Heart Alliance, Munich), Bernhard Krämer (University Clinic, Mannheim), Gerrit Kuprat (private practice, Rain), Ann-Cathrin Koschker (Lipid Clinic University Clinic, Würzburg), Bernhard Krämer (University Clinik, Mannheim), Özlem Kilic (private practice, Hamburg), Ulrich Laufs (University Clinic, Leipzig), Gerd Lindenmeier (Kliniken an der Paar - Hospital Friedberg), Iris van de Loo (Endokrinologikum Bremen), Babette Lorenz (Dialyse Hohenschönhausen), Elke Lorenz (German Heart Centre Munich), Birgit Löhr (University Clinic Augsburg), Johanna McChord (Robert-Bosch-Hospital, Stuttgart), Mariya Maslarska (University Heart Centre, Freiburg), Heiko Methe (Kliniken an der Paar - Hospital Aichach), Martin Merkel (Endokrinologikum, Hamburg), Zineb Moussaoui (Robert-Bosch-Hospital, Stuttgart), Irina Müller-Kozarez (University Clinic, Leipzig), Christoph B Olivier (University Heart Centre, Freiburg), Peter Ong (Robert-Bosch-Hospital, Stuttgart), Britta Otte (Medical Clinic D, Lipid Clinic, University Hospital Münster), Klaus Parhofer (Medical clinic and Polyclinic IV, Ludwig-Maximilian University, Munich), Carl-Joachim Partsch (Endokrinologikum, Hamburg), Michael Paulus (Lipid Clinic; Clinic and Policlinic for Internal Medicine II; University Clinic Regensburg), Sinan Pehlivanli (Kliniken an der Paar - Hospital Aichach), Tobias Pflederer (private practice, Kempten), Thomas Pusl (University Clinic Augsburg), Veronika Richter (private practice, Hannover), Stefanie Rosner (German Heart Centre Munich), Veronika Sanin (German Heart Centre Munich), Sebastian Schäfer (private practice, Köln Rodenkirchen), Christoph Schäfer (private practice, Köln Rodenkirchen), Ulrike Schatz (Dept. of Internal Medicine III, University Hospital Carl Gustav Carus at the Technische Universität Dresden), Stephan Schirmer (private practice, Kaiserslautern), Christine Schmidt (University Hospital Leipzig), Wolfgang Seeger (private practice, Berlin), Snezna Sisovic (Medical clinic and Polyclinic IV, Ludwig-Maximilian University, Munich), Antje Spens (MVZ Stoffwechselmedizin Leipzig), Ksenija Stach Jablonski (University Clinic, Mannheim), Alexander Stadelmann (private practice, Nürnberg), Elisabeth Steinhagen-Thiessen (Centre for Internal Medicine with Gastroenterology and Nephrology, Lipid Clinic, Charité Berlin), Paulina Stürzebecher (University Clinic, Leipzig), Maria Tafelmeier (Lipid Clinic; Clinic and Policlinic for Internal Medicine II; University Clinic Regensburg), Dörthe Tillack (private practice, Haslach im Kinzigtal), Sergey Tselmin (Dept. of Internal Medicine III, University Hospital Carl Gustav Carus at the Technische Universität Dresden), Adrienn Tünnemann-Tarr (Clinic and Policlinic for Cardiology, Dept. for Internal Medicine, Neurology and Dermatology, University Hospital Leipzig), Anja Vogt (Medical clinic and Polyclinic IV, Ludwig-Maximilian University, Munich), Jens von Beckerath (Cardiologikum, Hamburg), Andreas Wilke (private practice, Papenburg), Ulrich Wolf (private practice, Stahnsdorf), Claudia Zemmrich (private practice, Berlin). **GREECE:** Christos V. Rizos (Dept. of Internal Medicine, Faculty of Medicine, University of Ioannina, Ioannina), Ioannis Skoumas (1st Dept. of Cardiology, Medical School, National and Kapodistrian University of Athens, Hippokration Hospital, Athens), Konstantinos Tziomalos (1st Propedeutic Dept. of Internal Medicine, Medical School, Aristotle University of Thessaloniki, AHEPA Hospital, Thessaloniki), Loukianos Rallidis (Dept. of Cardiology, Medical School, National and Kapodistrian University of Athens, Attikon University General Hospital, Athens), Vasileios Kotsis (Dept. of Internal Medicine, Medical School, Aristotle University of Thessaloniki, Papageorgiou General Hospital Thessaloniki, Thessaloniki), Michalis Doumas (Dept. of Internal Medicine, Medical School, Aristotle University of Thessaloniki, Hippokration General Hospital, Thessaloniki), Vasileios Athyros (Dept. of Internal Medicine, Medical School, Aristotle University of Thessaloniki, Hippokration General Hospital, Thessaloniki), Emmanouil Skalidis (Cardiology Clinic, University General Hospital of Heraklion, Heraklion), Genovefa Kolovou (Metropolitan Hospital, Cardiometabolic Center, Lipoprotein Apheresis and Lipid Disorders Clinic, Athens, Greece), Vana Kolovou (Metropolitan Hospital, Cardiometabolic Center, Lipoprotein Apheresis and Lipid Disorders Clinic, Athens, Greece), Anastasia Garoufi (Dept. of Pediatrics, Medical School, National and Kapodistrian University of Athens, 2nd Pediatrics Clinic, General Children’s Hospital “Pan. & Aglaia Kyriakou”, Athens), Vasiliki Giannakopoulou (Cardiology Clinic, ‘Tzaneio’ General Hospital, Piraeus), Ioanna Dima(1st Dept. of Cardiology, Medical School, National and Kapodistrian University of Athens, Hippokration Hospital, Athens), Κonstantinos Papathanasiou (Dept. of Cardiology, Medical School, National and Kapodistrian University of Athens, Attikon University General Hospital, Athens), Christina Antza (Dept. of Internal Medicine, Medical School, Aristotle University of Thessaloniki, Papageorgiou General Hospital Thessaloniki, Thessaloniki), Evangelos Zacharis (Cardiology Clinic, University General Hospital of Heraklion, Heraklion), Achilleas Attilakos (Dept. of Pediatrics, Medical School, National and Kapodistrian University of Athens, C' Pediatrics Clinic, Attikon University General Hospital, Athens), George Sfikas (Dept. of Internal Medicine, 424 Military Hospital, Thessaloniki), Charalambos Koumaras (Dept. of Internal Medicine, 424 Military Hospital, Thessaloniki), Panagiotis Anagnostis (Dept. of Endocrinology, Police Medical Centre of Thessaloniki, Thessaloniki), Georgia Anastasiou (Dept. of Internal Medicine, Faculty of Medicine, University of Ioannina, Ioannina), George Liamis (Dept. of Internal Medicine, Faculty of Medicine, University of Ioannina, Ioannina), Amalia-Despoina Koutsogianni (Dept. of Internal Medicine, Faculty of Medicine, University of Ioannina, Ioannina), Ermioni Petkou (Dept. of Internal Medicine, Faculty of Medicine, University of Ioannina, Ioannina), Haralambos Milionis (Dept. of Internal Medicine, Faculty of Medicine, University of Ioannina, Ioannina), , Elisavet Prodromiadou (First Department of Propedeutic Medicine, School of Medicine, National and Kapodistrian University of Athens, Athens), Amalia Boufidou (1st Department of Cardiology, Medical School, Aristotle University of Thessaloniki, AHEPA Hospital, Thessaloniki, Greece), Vaia Lambadiari (2nd Propaedeutic Internal Medicine Department and Diabetes Research Unit, National and Kapodistrian University of Athens, Attikon University General Hospital, Athens, Greece), Kimon Stamatelopoulos (Department of Clinical Therapeutics, School of Medicine, National and Kapodistrian University of Athens, Athens, Greece). **HUNGARY:** Zsolt Karányi (Dept. of Internal Medicine, Faculty of Medicine, University of Debrecen), Mariann Harangi (Dept. of Internal Medicine, Faculty of Medicine, University of Debrecen), László Bajnok (1st Dept. of Medicine, University of Pécs, Pécs), Mária Audikovszky (Metabolic Centre, Szent Imre University Teaching Hospital, Budapest), László Márk (2nd Dept. of Medicine e Cardiology, Pandy Kalman Bekes County Hospital, Gyula), Béla Benczúr (1st Dept. of Internal Medicine Cardiology/Nephrology, Balassa János County Hospital, Szekszárd), István Reiber (Fejér County St György Teaching Hospital, Székesfehérvár), Gergely Nagy (Borsod-Abaúj-Zemplén County University Teaching Hospital, Miskolc), András Nagy (1st Dept. of Internal Medicine Bács-Kiskun County Hospital-Cardiology, Kecskemét). **INDIA:** Lakshmi Lavanya Reddy (P. D Hinduja Hospital and Medical Research Centre, Mahim, Mumbai), Swarup A. V Shah (P. D Hinduja Hospital and Medical Research Centre, Mahim, Mumbai), Chandrashekhar K. Ponde (P. D Hinduja Hospital and Medical Research Centre, Mahim, Mumbai), Jamshed J. Dalal (Kokilaben Dhidubhai Ambani Hospital and Medical Research Institute, Mumbai), Jitendra P. S. Sawhney (Sir Ganga Ram Hospital, New Delhi), Ishwar C. Verma (Sir Ganga Ram Hospital, New Delhi), Dr. Krishnakumar B. Pillai (Government Medical College, Cochin, Kerala). **INDONESIA:** Adrian Reynaldo Sudirman (Universitas Indonesia), Kelvin Supriami (Harvard University), Heniszayanti Nabiladhiya Asrialdi (Universitas Indonesia Hospital), Muhammad Naufal Zuhdi (National Cardiovascular Center Harapan Kita), Putra Andito Ramadhan (Universitas Indonesia). **IRAQ:** Ali Fawzi Abdalsahib (University of Al-Qadisiyah, College of medicine), Mohammed Dhamin (University of Kufa, Faculty of medicine) Sarmad AlFil (MOH, Alkhansaa Pediatric teaching hospital) Foad Kadhim (Jabir Ibin hayyan medical university). **IRELAND:** Ruth Agar (Tallaght University Hospital). **ITALY:** Alberico Luigi Catapano (IRCCS MultiMedica, Sesto San Giovanni - Milan); Marcello Arca (Dipartimento di Medicina Traslazionale e di Precisione Sapienza Università di Roma; A.O. Policlinico Umberto I, Rome), Maurizio Averna (Department of Health Promotion, Mother and Child Care, Internal Medicine and Medical Specialties (PROMISE), Università degli Studi di Palermo, Palermo; Istituto di Biofisica, Consiglio Nazionale delle Ricerche, Palermo), Stefano Bertolini (Department of Internal Medicine, University of Genoa, Genoa), Sebastiano Calandra (Department of Biomedical, Metabolic and Neural Sciences, University of Modena and Reggio Emilia, Modena), Patrizia Tarugi (Department of Life Sciences, University of Modena and Reggio Emilia, Modena), Manuela Casula (Epidemiology and Preventive Pharmacology Service SEFAP, Dept. of pharmacological and biomolecular sciences, University of Milan, Milan; IRCCS MultiMedica, Sesto San Giovanni - Milan), Federica Galimberti (IRCCS MultiMedica, Sesto San Giovanni - Milan), Marta Gazzotti (Fondazione SISA, Milan), Elena Olmastroni (Epidemiology and Preventive Pharmacology Service SEFAP, Dept. of pharmacological and biomolecular sciences, University of Milan, Milan), Riccardo Sarzani (Clinica di Medicina Interna e Geriatria, Dipartimento di Scienze Cliniche e Molecolari, Università Politecnica delle Marche; IRCCS-INRCA, Ancona), Claudio Ferri (Centro Ipertensione Arteriosa e Prevenzione, Cardiovascolare; UOC Medicina Interna e Nefrologia, Università dell’Aquila; Dipartimento MeSVA, Università dell’Aquila; Ospedale San Salvatore, L'Aquila), Elena Repetti (SOC Diabetologia e Malattie metaboliche, ASL AT, Asti), Francesco Giorgino (Department of Precision and Regenerative Medicine and Ionian Area, Section of Internal Medicine, Endocrinology, Andrology and Metabolic Diseases, University of Bari Aldo Moro, Bari); Patrizia Suppressa (U.O. di Medicina Interna e Geriatria “C. Frugoni” e Centro di Assistenza e Ricerca Malattie Rare, A.O. Universitaria Policlinico Consorziale, Università degli Studi di Bari "Aldo Moro", Bari), Giancarla Meregalli (U.O.C. Malattie Endocrine e Centro regionale per il Diabete Mellito, ASST Bergamo Ovest, Treviglio), Claudio Borghi (U.O. di Medicina Interna Cardiovascolare, Università di Bologna; IRCCS S. Orsola Ospedale Policlinico S. Orsola-Malpighi, Bologna), Sandro Muntoni (Dipartimento di Scienze Biomediche, Università degli Studi di Cagliari; Centro per le Malattie Dismetaboliche e l’arteriosclerosi, Associazione ME.DI.CO Onlus, Cagliari), Paolo Calabrò (UOC Cardiologia Clinica a Direzione Universitaria e UTIC, AORN "Sant'Anna e San Sebastiano", Caserta; Dipartimento di Scienze Mediche Traslazionali Università degli studi della Campania "Luigi Vanvitelli", Naples), Francesco Cipollone (Clinica Medica, Centro di alta specializzazione per la prevenzione dell’arteriosclerosi centro di eccellenza ESH per l’ipertensione arteriosa, centro di riferimento regionale per le Dislipemie, Ospedale Policlinico S.S. Annunziata, Chieti), Francesco Purrello (Department of Clinical and Experimental Medicine, University of Catania, Ospedale Garibaldi, Catania), Arturo Pujia (Dipartimento Scienze Mediche Chirurgiche, Università degli Studi Magna Graecia, Catanzaro), Angelina Passaro (Centro per lo Studio e il Trattamento delle Malattie del Metabolismo, Aterosclerosi e Nutrizione Clinica, Azienda Ospedaliera-Universitaria S. Anna di Ferrara; Dipartimento di Medicina Traslazionale e per la Romagna - Università degli Studi di Ferrara, Ferrara), Rossella Marcucci (Dipartimento medicina sperimentale e clinica, Università di Firenze, AOUC Azienda Ospedaliero-Universitaria Careggi, Firenze), Valerio Pecchioli (UOSD ‘Prevenzione cardiovascolare’, Dipartimento di Scienze Mediche, Azienda Sanitaria Locale Frosinone, Frosinone), Livia Pisciotta (IRCCS Ospedale policlinico San Martino UOSD Dietetica e Nutrizione Clinica, Dipartimento di Medicina Interna, Università di Genova, Genova), Giuseppe Mandraffino (Dipartimento di Medicina Clinica e Sperimentale - Centro per la Diagnosi e Cura della Dislipidemia e Prevenzione dell’Aterosclerosi, A.O. Universitaria Policlinico “G. Martino”, Messina), Fabio Pellegatta (Centro per lo Studio dell'Aterosclerosi, IRCCS MultiMedica, Sesto San Giovanni - Milan), Giuliana Mombelli (Centro Dislipidemie, ASST Grande Ospedale Metropolitano Niguarda, Milano), Adriana Branchi (Ambulatorio Dislipidemie, Centro per lo studio e la prevenzione dell'Arteriosclerosi , Fondazione IRCCS Ca’ Granda, Ospedale Maggiore Policlinico di Milano; Dipartimento di Scienze Cliniche e di Comunità, Università degli Studi di Milano, Milano), Anna Maria Fiorenza (ASST-Rhodense Garbagnate Milanese, Garbagnate Milanese), Cristina Pederiva (U.O. Clinica Pediatrica, Servizio clinico dislipidemie per lo studio e la prevenzione dell’aterosclerosi in età pediatrica, ASST-Santi Paolo e Carlo, Milano), Josè Pablo Werba (U.O. Ambulatorio Prevenzione Aterosclerosi, IRCCS Cardiologico Monzino, Milano), Gianfranco Parati (Istituto Auxologico Italiano, IRCCS Ospedale San Luca, Milan, Italy; Department of Medicine and Surgery, University of Milano-Bicocca, Milan), Francesca Carubbi (U.O. Medicina interna ad indirizzo metabolico-nutrizionistico, Centro dislipidemie e malattie metaboliche rare, Ospedale Civile Baggiovara, AOU di Modena, Modena), Lorenzo Iughetti (U.O.C. Pediatria, Azienda Ospedaliero Universitaria di Modena, Modena), Giuliana Fortunato (Dipartimento di Medicina Molecolare e Biotecnologie Mediche, Università degli studi di Napoli Federico II, Naples; CEINGE Biotecnologie Avanzate scarl Naples,); Arcangelo Iannuzzi (U.O. Medicina Interna 2, Centro per le malattie da arteriosclerosi, AORN Cardarelli, Napoli), Gabriella Iannuzzo (Dipartimento di Medicina Clinica e Chirurgia, Università degli Studi di Napoli Federico II, Naples), Angelo Baldassare Cefalù (Department of Health Promotion, Mother and Child Care, Internal Medicine and Medical Specialties (PROMISE), Università degli Studi di Palermo, Palermo), Giacomo Biasucci (Centro Dislipidemie in Età evolutiva, U.O. Pediatria e Neonatologia, Ospedale Guglielmo da Saliceto, Piacenza, Piacenza), Sabina Zambon (U. O. Clinica Medica 1, Centro Dislipidemie e Aterosclerosi, A.O. di Padova, Padova), Matteo Pirro (Sezione Medicina Interna, Angiologia e Malattie da Arteriosclerosi, Dipartimento di Medicina e Chirurgia, Università degli Studi di Perugia, Perugia), Francesco Sbrana (U.O. Lipoaferesi, Centro Regionale di Riferimento per la diagnosi e cura delle Dislipidemie Ereditarie, Fondazione Toscana "G. Monasterio", Pisa); Chiara Trenti (Arcispedale S. Maria Nuova - Azienda Ospedaliera di Reggio Emilia, Reggio Emilia), Laura D’Erasmo (Dipartimento di Medicina Traslazionale e di Precisione Sapienza Università di Roma, Roma), Massimo Federici (Dipartimento di Medicina dei Sistemi, Università di Roma Tor Vergata, Roma), Maria Del Ben (Dipartimento Scienze Cliniche, Internistiche, Anestesiologiche e Cardiovascolari - Sapienza Università, A.O. Policlinico Umberto I, Roma), Andrea Bartuli (UO Malattie Rare e Genetica Medica, Ospedale Pediatrico Bambino Gesù, IRCCS, Roma), Andrea Giaccari (UOC Endocrinologia e Malattie del Metabolismo, Fondazione Policlinico Universitario A. Gemelli IRCCS, Roma), Antonio Pipolo (AOU San Giovanni di Dio e Ruggi d'Aragona, Salerno), Nadia Citroni (Centro Dislipidemie e Aterosclerosi, UOC Medicina Interna, Ospedale di Trento, Trento), Ornella Guardamagna (Paediatric Endocrinology, Department of Public Health and Paediatric Sciences, Turin University, Turin), Salvatore Lia (AOU San Luigi Gonzaga, Orbassano), Andrea Benso (SCDU Endocrinologia, Diabetologia e Malattie del Metabolismo, Dipartimento di Scienze Mediche, Università degli Studi di Torino, Torino), Gianni Biolo (SS di Diabetologia e Malattie Metaboliche, U.C.O. Clinica Medica, ASUGI, Università di Trieste, Trieste), Lorenzo Maroni (Ambulatorio ipertensione dislipidemie, U.O. Medicina Generale, ASST Valle Olona, Ospedale di Gallarate, Gallarate), Alessandro Lupi (ASL VCO - UO SOC Cardiologia, Ospedale Castelli, Verbania), Luca Bonanni (Ambulatorio Dislipidemie UO Medicina Interna Ospedale dell'Angelo di Mestre, Venezia), Elisabetta Rinaldi (U.O. Endocrinologia Diabetologia e Malattie del Metabolismo, Centro regionale specializzato per la diagnosi e terapia delle dislipidemie e aferesi terapeutica, A.O. Universitaria Integrata di Verona, Verona) Maria Grazia Zenti (Servizio di Diabetologia e Malattie Metaboliche "Ospedale P. Pederzoli", Casa di Cura Privata SpA., Peschiera del Garda). **JAPAN:** Kota Matsuki (National Cerebral and Cardiovascular Centre Research Institute), Mika Hori (National Cerebral and Cardiovascular Centre Research Institute), Masatsune Ogura (National Cerebral and Cardiovascular Centre Research Institute), Daisaku Masuda (Rinku General Medical Centre), Takuya Kobayashi (Rinku General Medical Centre), Kumiko Nagahama (Rinku General Medical Centre). **KUWAIT:** Mohammed Al-Jarallah (Sabah Al Ahmad Cardiac Centre), Mirjana Radovic (Sabah Al Ahmad Cardiac Centre). **KYRGYZSTAN:** Olga Lunegova (Kyrgyz State Medical Academy), Erkayim Bektasheva (Kyrgyz State Medical Academy), Saamay Abilova (Kyrgyz State Medical Academy). **LATVIA:** Andrejs Erglis (Research Institute of Cardiology and Regenerative Medicine, Faculty of Medicine, University of Latvia, Pauls Stradins Clinical University Hospital, Riga), Dainus Gilis (Research Institute of Cardiology and Regenerative Medicine, Faculty of Medicine, University of Latvia, Pauls Stradins Clinical University Hospital, Riga), Georgijs Nesterovics (Research Institute of Cardiology and Regenerative Medicine, Faculty of Medicine, University of Latvia, Pauls Stradins Clinical University Hospital, Riga), Vita Saripo (Research Institute of Cardiology and Regenerative Medicine, University of Latvia, Pauls Stradins Clinical University Hospital, Riga), Ruta Meiere (Research Institute of Cardiology and Regenerative Medicine, University of Latvia, Pauls Stradins Clinical University Hospital, Riga), Gunda Skudrina (Research Institute of Cardiology and Regenerative Medicine, University of Latvia, Pauls Stradins Clinical University Hospital, Riga), Elizabete Terauda (Research Institute of Cardiology and Regenerative Medicine, University of Latvia, Pauls Stradins Clinical University Hospital, Riga). **LEBANON:** Selim Jambart (Saint Joseph University of Beirut Faculty of Medicine, Hôtel Dieu Hospital, Beirut), Carine Ayoub (Laboratory of Biochemistry and Molecular Therapeutics, Faculty of Pharmacy, Saint Joseph University, Beirut), Youmna Ghaleb (Laboratory of Biochemistry and Molecular Therapeutics, Faculty of Pharmacy, Saint Joseph University, Beirut). **LITHUANIA:** Urte Aliosaitiene (Vilnius University Faculty of Medicine, Vilnius, Lithuania; Vilnius University Hospital Santaros Klinikos, Vilnius), Sandra Kutkiene (Vilnius University Faculty of Medicine, Vilnius, Lithuania; Vilnius University Hospital Santaros Klinikos, Vilnius). **MALAYSIA:** Siti Hamimah Sheikh Abdul Kadir (Institute of Pathology, Laboratory and Forensic Medicine [I-PPerForM] and Faculty of Medicine, Universiti Teknologi MARA (UiTM) Noor Alicezah Mohd Kasim (Hospital Al-Sultan Abdullah [HASA] Teaching Hospital and Faculty of Medicine, UiTM , Noor Shafina Mohd Nor (HASA Teaching Hospital and Faculty of Medicine, UiTM), Hasidah Abdul Hamid (Faculty of Medicine, UiTM), Suraya Abdul Razak (HASA Teaching Hospital and Faculty of Medicine, UiTM), Alyaa Al-Khateeb (I-PPerForM and Faculty of Medicine, UiTM), Suhaila Abd Muid (I-PPerForM and Faculty of Medicine, UiTM), Thuhairah Abdul Rahman (HASA Teaching Hospital and Faculty of Medicine, UiTM), Sazzli Shahlan Kasim (HASA Teaching Hospital and Faculty of Medicine, UiTM), Ahmad Bakhtiar Md Radzi (HASA Teaching Hospital and Faculty of Medicine, UiTM), Khairul Shafiq Ibrahim (HASA Teaching Hospital and Faculty of Medicine, UiTM), Marshima Mohd Rosli (Faculty of Information Technlogy and Computer Science, UiTM), Rafezah Razali (HASA Teaching Hospital), Yung An Chua (I-PPerForM and Faculty of Medicine, UiTM), Aimi Zafira Razman (I-PPerForM UiTM), Sukma Azureen Nazli (I-PPerForM UiTM), Nazirul Aziz ((I-PPerForM UiTM), Azhari Rosman (National Heart Institute, Kuala Lumpur), NorAzian Abdul Murad (Universiti Kebangsaan Malaysia) Mohd Amin Jalaludin, (University of Malaya), Ahmad Zubaidi Abdul Latif (UniSZA). **MALTA:** C. Azzopardi (University of Malta Medical School, Dept. of Medicine; Lipid Clinic, Mater Dei Hospital). **MEXICO:** Roopa Mehta (Unidad de Investigación de Enfermedades Metabolicas and Departamento de Endocrinologia y Metabolismo, Instituto Nacional de Ciencias Médicas y Nutrición Salvador Zubiran, México City), Alexandro J. Martagon (Unidad de Investigación de Enfermedades Metabólicas, Instituto Nacional de Ciencias Médicas y Nutrición Salvador Zubirán, Mexico City, México and Tecnologico de Monterrey, Escuela de Medicina y Ciencias de la Salud, Monterrey, N.L., México), Gabriela A. Galan Ramirez (Unidad de Investigación de Enfermedades Metabólicas, Instituto Nacional de Ciencias Médicas y Nutrición Salvador Zubirán, Mexico City, México), Neftali E Antonio Villa (Faculty of Medicine, Universidad Nacional Autónoma de México. Mexico), Arsenio Vargas Vazquez (Faculty of Medicine, Universidad Nacional Autónoma de México. Mexico), Daniel Elias-Lopez (Unidad de Investigación de Enfermedades Metabolicas and Departamento de Endocrinologia y Metabolismo, Instituto Nacional de Ciencias Médicas y Nutrición Salvador Zubiran, México City), Gustavo Gonzalez Retana (Departamento de Endocrinologia y Metabolismo, Instituto Nacional de Ciencias Médicas y Nutrición Salvador Zubiran, México City), Betsabel Rodriguez (Departamento de Endocrinologia y Metabolismo, Instituto Nacional de Ciencias Médicas y Nutrición Salvador Zubiran, México City), Jose J. Ceballos Macías (Secretaria de la Defensa Nacional SEDENA, Mexico City), Alejandro Romero Zazueta (Hospital Angeles, Culiacan,Sinaloa), Rocio Martinez Alvarado (Instituto Nacional de Cardiologia – Ignacio Chavez, Mexico City), Julieta D. Morales Portano (Centro Médico Nacional 20 de Noviembre ISSSTE, Mexico City), Humberto Alvares Lopez (Torre Medyca, Saltillo, Coahuila), Leobardo Sauque-Reyna (Instituto de Diabetes Obesidad y Nutricion S.C., Cuernavaca, Morelos), Laura G. Gomez Herrera (Hospital General Zona #2 IMSS, Saltillo, Coahuila), Luis E. Simental Mendia (Instituto Mexicano del Seguro Social (IMSS), Durango), Humberto Garcia Aguilar (Hospital Angeles Lomas, Estado de Mexico), Elizabeth Ramirez Cooremans (Clinica Condomedics, Torreon, Coahuila), Berenice Peña Aparicio (Instituto Nacional de Cardiologia – Ignacio Chavez, Mexico City), Victoria Mendoza Zubieta (Centro Médico Nacional S. XXI, Instituto Mexicano del Seguro Social, Mexico City), Perla A. Carrillo Gonzalez (Hospital Regional Lic. Adolfo Lopez Mateos, ISSSTE, Mexico City), Aldo Ferreira-Hermosillo (Centro Médico Nacional S. XXI, Instituto Mexicano del Seguro Social, Mexico City), Nacu Caracas Portilla (Instituto Nacional de Cardiologia – Ignacio Chavez, Mexico City), Guadalupe Jimenez Dominguez (Hospital General Zona #46 IMSS, Villahermosa and Hospital Angeles de Villahermosa, Tabasco), Alinna Y. Ruiz Garcia (Hospital AMERIMED, Cancun), Hector E. Arriaga Cazares (Centro Médico Nacional del Noreste IMSS, Monterrey and Tecnologico de Monterrey, Escuela de Medicina y Ciencias de la Salud, Monterrey, N.L., México), Jesus R. Gonzalez (Hospital de Cardiologia de Aguascalientes, Aguascalientes), Carla V. Mendez Valencia (Unidad Médica de Alta Especialidad 2 (UMAE), IMSS, Ciudad Obregon Sonora), Francisco G. Padilla (Cardiologia Clinica e Intervencionista, Guadalajara, Jalisco), Ramon Madriz Prado (Secretaria de la Defensa Nacional (SEDENA), Mexico City), Manuel O. De los Rios Ibarra (SINACOR, Culiacan, Sinaloa), Ruy D. Arjona Villicaña (Hospital Regional de Alta Especialidad, Merida, Yucatan), Karina J. Acevedo Rivera (Unidad de Endocrinolgia, Tijuana, Baja California), Ricardo Allende Carrera (Hospital de Especialidades Médicas de la Salud, San Luis Potosi), Jose A. Alvarez (Hospital Central Norte de PEMEX, Mexico City), Jose C. Amezcua Martinez (Hospital de Especialidades El Angel, Uruapan, Michoacan), Manuel de los Reyes Barrera Bustillo (Clinica de Merida, Yucatan), Gonzalo Carazo Vargas (Instituto Nacional de Cardiologia – Ignacio Chavez, Mexico City), Roberto Contreras Chacon (Instituto Cardiovascular de Penjamo, Guanajuato), Mario H. Figueroa Andrade (Hospital General de Zona 1, Colima), Ashanty Flores Ortega (Servicios de Salud del Estado de Hidalgo, Pachuca, Hidalgo), Hector Garcia Alcala (Hospital Christus Muguerza UPAEP, Puebla), Laura E. Garcia de Leon (Centro Hospitalario La Concepcion, Saltillo, Coahuila), Berenice Garcia Guzman (Hospital Angeles del Pedregal, Mexico City), Jose J. Garduño Garcia (UAE Mexico – Hospital General Regional #251, IMSS, Toluca, Estado de Mexico), Juan C. Garnica Cuellar (Hospital de Especialidades del Centro Médico Nacional – La Raza, IMSS, Mexico City), Jose R. Gomez Cruz (Hospital Angeles de Xalapa, Veracruz, Mexico), Anell Hernandez Garcia (Clinica , Oaxaca), Jesus R. Holguin Almada (Casa del diabetico Sonora, Mexico), Ursulo Juarez Herrera (Instituto Nacional de Cardiologia – Ignacio Chavez, Mexico City), Fabiola Lugo Sobrevilla (IMSS, Reynosa, Tamaulipas), Eduardo Marquez Rodriguez (Instituto Jaliscience de Metabolismo, Guadalajara, Jalisco), Cristina Martinez Sibaja (Facultad de Medicina Miguel Aleman, Veracruz), Alma B. Medrano Rodriguez (Torre Medyca, Saltillo, Coahuila), Jose C. Morales Oyervides (Hospital General Regional IMSS 270, Reynosa, Tamaulipas), Daniel I. Perez Vazquez (Smart Heart, Cholula Puebla), Eduardo A. Reyes Rodriguez (Hospital Regional PEMEX, Ciudad Madero, Tamaulipas), Ma. Ludivina Robles Osorio (Universidad Autónoma de Querétaro, Queretaro), Juan Rosas Saucedo (Instituto de Diabetes de Celaya, Guanajuato), Margarita Torres Tamayo (Instituto Nacional de Cardiologia – Ignacio Chavez, Mexico City), Luis A. Valdez Talavera (Centro Hospitalario La Concepcion, Saltillo, Coahuila), Luis E. Vera Arroyo (Centro el Corazon de Coahuila, Torreon, Coahuila), Eloy A. Zepeda Carrillo (Hospital Civil Tepic, Nayarit). **NETHERLANDS:** L Zuurbier (Department of Clinical Genetics, Amsterdam UMC Location AMC, Meibergdreef 9, 1105, AZ, Amsterdam), L. Reeskamp (Department of Vascular Medicine, Amsterdam UMC Location AMC, Meibergdreef 9, 1105, AZ, Amsterdam), S Ibrahim (Department of Vascular Medicine, Amsterdam UMC Location AMC, Meibergdreef 9, 1105, AZ, Amsterdam), J Roeters van Lennep (Department of Internal Medicine, Erasmus University Medical Center, Rotterdam), A Wiegman (Department of Paediatrics, Amsterdam UMC location University of Amsterdam, Amsterdam). **NIGERIA:** Vivienne Omuemu (Dept. Of Community Health, University of Benin Teaching Hospital, Benin City, Edo State), Alphonsus Isara (Dept. of Community Health, University of Benin Teaching Hospital, Benin City, Edo State), Darlington E. Obaseki (Dept. of Pathology, University of Benin Teaching Hospital, Benin City, Edo State), Vivienne Omuemu (Dept. Of Community Health, University of Benin Teaching Hospital, Benin City, Edo State). **NORWAY:** Martin P. Bogsrud, Unit for Cardiac and Cardiovascular genetics, Oslo University Hospital, Oslo, Norway. **OMAN:** Khalid Al-Waili (Dept. of Biochemistry, Sultan Qaboos University Hospital, Muscat), Fahad Al-Zadjali (Dept. of Biochemistry, College of Medicine & Health Science, Sultan Qaboos University, Muscat), Ibrahim Al-Zakwani (Dept. of Pharmacology, College of Medicine & Health Science, Sultan Qaboos University, Muscat), Mohammed Al-Kindi (Dept. of Biochemistry, College of Medicine & Health Science, Sultan Qaboos University, Muscat), Suad Al-Mukhaini (Dept. of Nursing, Sultan Qaboos University Hospital, Muscat), Hamida Al-Barwani (Dept. of Biochemistry, College of Medicine & Health Science, Sultan Qaboos University, Muscat). **PAKISTAN:** Asim Rana (Dept. of Medicine & Critical Care, Bahria International Hospital, Lahore), Hijab Batool (Chemical Pathology, Chugtai Institute of Pathology, Lahore), Saeed Ullah Shah, Munir Malik, Sabeen Khan (Shifa International Hospital, Islamabad), Arshad Hussain (NorthWest General Hospital & Research Centre, Passport Office Road, Hayatabad, Peshawer) **POLAND:** Ewa Starostecka (Regional Centre for Rare Diseases, Polish Mother’s Memorial Hospital Research Institute PMMHRI, Lodz), Agnieszka Konopka (Regional Centre for Rare Diseases, Polish Mother’s Memorial Hospital Research Institute PMMHRI, Lodz), Joanna Lewek (Dept. of Preventive Cardiology and Lipidology, Medical University of Lodz; Polish Mother’s Memorial Hospital Research Institute PMMHRI, Lodz), Bozena Sosnowska (Dept. of Preventive Cardiology and Lipidology, Medical University of Lodz; Polish Mother’s Memorial Hospital Research Institute PMMHRI, Lodz), Agata Bielecka-Dabrowa (Dept. of Cardiology and Congenital Diseases of Adults, Polish Mother’s Memorial Hospital Research Institute PMMHRI, Lodz), Mariusz Gąsior (3rd Dept. of Cardiology, School of Medicine with the Division of Dentistry in Zabrze, Medical University of Silesia, Katowice; Silesian Centre for Heart Diseases in Zabrze), Krzysztof Dyrbuś (3rd Dept. of Cardiology, School of Medicine with the Division of Dentistry in Zabrze, Medical University of Silesia, Katowice; Silesian Centre for Heart Diseases in Zabrze), Jacek Jóźwiak (Dept. of Family Medicine and Public Health, Institute of Medicine, University of Opole, Opole), Marcin Pajkowski (National Centre of Familial Hypercholesterolaemia, Dept. of Cardiac, Diagnostics, 1-st Dept. of Cardiology, Medical University of Gdansk), Marzena Romanowska-Kocejko (National Centre of Familial Hypercholesterolaemia, University Clinical Centre in Gdańsk), Marta Żarczyńska-Buchowiecka (National Centre of Familial Hypercholesterolaemia, Dept. of Cardiac Diagnostics, Medical University of Gdansk), Magdalena Chmara (Dept. of Biology and Medical Genetics Medical University of Gdańsk), Bartosz Wasąg (Dept. of Biology and Medical Genetics Medical University of Gdańsk), Aneta Stróżyk (National Centre of Familial Hypercholesterolaemia, University Clinical Centre in Gdańsk), Aleksandra Michalska-Grzonkowska (National Centre of Familial Hypercholesterolaemia, University Clinical Centre in Gdańsk). **PORTUGAL:** Ana Margarida Medeiros (Instituto Nacional de Saúde Doutor Ricardo Jorge, Departamento de Promoção da Saúde e Prevenção de Doenças Não Transmissíveis Unidade de I&D, Grupo de Investigação Cardiovascular, Lisboa), Ana Catarina Alves (Instituto Nacional de Saúde Doutor Ricardo Jorge, Departamento de Promoção da Saúde e Prevenção de Doenças Não Transmissíveis Unidade de I&D, Grupo de Investigação Cardiovascular, Lisboa), Francisco Silva (Hospital Dr. Nélio Mendonça, Serviço de Pediatria, Funchal), Goreti Lobarinhas (Hospital Santa Maria Maior Barcelos, Serviço de Pediatria), Isabel Palma (Centro Hospitalar Universitário do Porto, Hospital de Sto Antonio, Serviço Endocrinologia), Jose Pereira de Moura (Centro Hospitalar e Universitário de Coimbra, Serviço Medicina Interna), Miguel Toscano Rico (Centro Hospitalar Lisboa Central, Hospital de Sta Marta, Serviço Medicina Interna), Quitéria Rato (Centro Hospitalar de Setúbal, Hospital S. Bernardo, Serviço de Cardiologia), Patrícia Pais (Centro Hospitalar do Barreiro Montijo, Serviço de Pediatria), Susana Correia (Centro Hospitalar do Barreiro Montijo, Serviço de Pediatria), Oana Moldovan (Centro Hospitalar de Lisboa Norte, Hospital de Sta. Maria, Serviço de Genética Médica), Maria João Virtuoso (Centro Hospitalar do Algarve - Faro, Serviço de Pediatria), Francisco Araujo (Hospital Lusiadas, Departamento de Medicina Interna), Jose Miguel Salgado (Hospital Senhora da Oliveira, Serviço de Pediatria), Inês Colaço (Centro Hospitalar Universitario Lisboa Norte, Hospital de Sta Maria, Serviço Medicina Interna). **RUSSIA:** Alexey Meshkov (Federal State Institution, National Medical Research Centre for Therapy and Preventive Medicine, of the Ministry of Health of the Russian Federation Moscow), Alexandra Ershova (Federal State Institution, National Medical Research Centre for Therapy and Preventive Medicine of the Ministry of Health of the Russian Federation Moscow), Tatiana Rozhkova (Federal State Institution National Medical Research Centre of cardiology n.a. Acad. E.I. Chazov, Ministry of Health of the Russian Federation, Moscow), Victoria Korneva (Petrozavodsk State university, faculty therapy Dept., Pertozavodsk. North-Western Region), Kuznetsova T. Yu (Petrozavodsk State university, faculty therapy Dept., Pertozavodsk, North-Westen Regio), Vitaliy Zafiraki (Kuban State Medical University Krasnodar City. South Region), Mikhail Voevoda (Federal Research Centre of fundamental and translational medicine, Novosibirsk), Victor Gurevich (Centre of Atherosclerosis and Lipid Disorders, Saint-Petersburg State University; North-Western State Medical University named after I.I. Mechnikov, Saint-Petersburg), Dmitry Duplyakov (Samara Regional Cardiology Dispensary, Samara), Yulia Ragino (Research Institute of Internal and Preventive Medicine-Branch of Institute of Cytology and Genetics, Siberian Branch of Russian Academy of Sciences, Novosibirsk), Uliana Chubykina (National Medical Research Centre of Cardiology n.a. Acad. E.I. Chazov, Ministry of Health of the Russian Federation, Moscow), Igor Shaposhnik (South-Ural State Medical University of the Ministry of Health of the Russian Federation, Chelyabinsk). **SAUDI ARABIA:** Alia Khudari (Cardiovascular Prevention Unit. Adult Cardiology Dept. Prince Sultan Cardiac Centre Riyadh), Nawal Rwaili (Cardiovascular Prevention Unit. Adult Cardiology Dept. Prince Sultan Cardiac Centre Riyadh), Faisal Al-Allaf (Dept. of Medical Genetics, Faculty of Medicine, Umm Al-Qura University, Makkah. Saudi Arabia), Mohammad Alghamdi (National Guard Hospital, Riyadh), Mohammed A Batais (Dept. of Family and Community Medicine, College of Medicine, King Saud University, Riyadh), Turky H Almigbal (Dept. of Family and Community Medicine, College of Medicine, King Saud University, Riyadh), Abdulhalim Kinsara (King Saud bin Abdulaziz University for Health Sciences, College of Medicine, King Abdul Aziz Medical City, Jeddah), Ashraf Hammouda Ahmed AlQudaimi (Saud Al Babtain Cardiac Centre, Dammam), Zuhier Awan (King Abdulaziz University, Jeddah), Omer A Elamin (King Faisal Specialist Hospital & Research Centre, Jeddah), Hani Altaradi (Dept. of Cardiac Sciences, King Fahad Cardiac Centre, College of Medicine, King Saud University, Riyadh). **SERBIA:** Ljiljana Popovic (Faculty of Medicine University of Belgrade, Clinic for Endocrinology, Diabetes and Metabolic Diseases, Belgrade), Sandra Singh (Faculty of Medicine University of Belgrade, Clinic for Endocrinology, Diabetes and Metabolic Diseases, Belgrade), Iva Rasulic (Faculty of Medicine University of Belgrade, Clinic for Endocrinology, Diabetes and Metabolic Diseases, Belgrade), Ana Petakov (Clinic for Endocrinology, Diabetes and Metabolic Diseases, Belgrade), Nebojsa M. Lalic (Faculty of Medicine University of Belgrade, Clinic for Endocrinology, Diabetes and Metabolic Diseases, Belgrade). **SINGAPORE:** Carolyn Lam (National Heart Centre), Tan Ju Le (National Heart Centre), Eric Lim Tien Siang (National Heart Centre), Sanjaya Dissanayake (Khoo Teck Puat Hospital), Justin Tang I-Shing (Khoo Teck Puat Hospital), Tai E Shyong (National University Hospital and NUS Saw Swee Hock School of Public Health), Terrance Chua Siang Jin (National Heart Centre, Singhealth), Sharon Pek Li Ting (Khoo Teck Puat Hospital), Jeremy Hoe Kian Ming (Khoo Teck Puat Hospital and Admiralty Medical Center), Chester Lee Drum (National University Hospital), Fathima Ashna Nastar (National University Hospital), Loh Wann Jia (Changi General Hospital), Natalie Koh Si Ya (Seng Kang Hospital), Marvin Chua Wei Jie (Seng Kang Hospital), Rinkoo Dalan (Tan Tock Seng Hospital), Yong Quek Wei (Tan Tock Seng Hospital), Tiong Yee sian (Ng Teng Fong General Hospital), Yeo Khung Keong (National Heart Centre Singapore), Siau Kai Rong (National University Polyclinics), Darren Seah Ee Jin (National Healthcare Group Polyclinics), Ian Koh Jan Ming (National Healthcare Group Polyclinics), Tan Hong Chang (Singapore General Hospital), Fabian Yap Kok Peng (KK Women’s and Children’s Hospital), Rashida Farhad Vasanwala (KK Women’s and Children’s Hospital). **SLOVAKIA:** Katarina Raslova (Coordination Centre for Familial Hyperlipidemias, Slovak Medical University in Bratislava), Karin Balinth (Dept. of Internal Medicine, Hospital Levice), Ingrid Buganova (Diabetes Clinic, MEDIVASA Ltd, Žilina), Lubomira Fabryova (Metabolklinik Ltd, Bratislava), Michaela Kadurova (Lipid Clinic, Poprad), Alexander Klabnik (Cardiology Clinic, Námestovo), Miriam Kozárová (IVth Dept. of Internal Medicine, Medical Faculty, PJ Šafárik University, Košice), Jana Sirotiakova (Dept. of Internal Medicine, Hospital Myjava). **SLOVENIA:** Tadej Battelino (UMC – University Children’s Hospital Ljubljana; University of Ljubljana, Faculty of Medicine), Matija Cevc (UMC Ljubljana), Marusa Debeljak (UMC – University Children’s Hospital Ljubljana; University of Ljubljana, Faculty of Medicine); Ana Drole Torkar (UMC – University Children’s Hospital Ljubljana; University of Ljubljana, Faculty of Medicine);  Zlatko Fras (UMC Ljubljana; University of Ljubljana, Faculty of Medicine); Borut Jug (UMC Ljubljana; University of Ljubljana, Faculty of Medicine), Barbara Kern Cugalj (UMC – University Children’s Hospital Ljubljana), Jernej Kovac (UMC – University Children’s Hospital Ljubljana; University of Ljubljana, Faculty of Medicine), Matej Mlinaric (UMC – University Children’s Hospital Ljubljana; University of Ljubljana, Faculty of Medicine), Katarina Trebusak Podkrajsek (UMC – University Children’s Hospital Ljubljana; University of Ljubljana, Faculty of Medicine, Ljubljana), Jaka Sikonja (UMC Ljubljana). **SOUTH AFRICA:** Gillian Joan Pilcher (Carbohydrate and Lipid Metabolism Research Unit, Faculty of Health Sciences, University of the Witwatersrand), D J Blom (University of Cape Town), K H Wolmarans (University of Cape Town), B C Brice (University of Cape Town). **SPAIN:** Ovidio Muñiz-Grijalvo (Dept. of Internal Medicine, Hospital Virgen del Rocío, Sevilla), Jose Luis Díaz-Díaz (Dept. of Internal Medicine, Hospital Abente y Lago, A Coruña), Leopoldo Pérez de Isla (Dept. of Cardiology, Hospital Clínico San Carlos, IDISSC, Madrid) Francisco Fuentes (Lipids and Atherosclerosis Unit, IMIBIC/Reina Sofia University Hospital, Cordoba), Lina Badimon (Programa-ICCC Cardiovascular, Institut de Recerca del Hospital Santa Creu i Sant Pau, IIB, Barcelona). **SWITZERLAND:** François Martin (diagene Research Institute, Kaegenstrasse, Reinach), Eleonore B. Miserez (diagene Research Institute, Kaegenstrasse, Reinach) Janine L. Shipton (Swiss Society of Familial Forms of Hypercholesterolemia). **THAILAND:** Poranee Ganokroj, Pairoj Chattranukulchai, Wiroj Jiamjarasrungsi (Chulalongkorn University and King Chulalongkorn Memorial Hospital), Nuntakorn Thongtang, Rungroj Krittayaphong (Siriraj Hospital, Mahidol University), Prin Vathesatogkit, Chutintorn Sriphrapradang (Ramathibodi Hospital, Mahidol University), Mattabhorn Phimphilai (Maharaj Nakorn Chiang Mai Hospital, Chiang Mai University), Rattana Leelawattana (Songklanagarind Hospital, Prince of Songkla University), Pimjai Anthanont (Thammasat Hospital, Thammasat University), Swangjit Suraamornkul (Vajira Hospital, Navamindradhiraj University), Chaicharn Deerochanawong (Rajavithi Hospital, Rangsit University), Vichai Senthong (Srinagarind Hospital, Khon Kaen University), Artit Torpongpun, Panuwat Suteerayongprasert (Chonburi Hospital), Nawarat Pengpong (King Prajadhipok Memorial Hospital), Nattapol Sathavarodom (Phramongkutklao Hospital and Phramongkutklao College of Medicine), Usanee Sunanta (Bhumibol Adulyadej Hospital), Thachanun Porntharukchareon (Chulabhorn Hospital, Chulabhorn Royal Academy), Phatharaporn Kiatpanabhikul (Charoenkrung Pracharak Hospital), Chatchon Kaewkrasaesin (Taksin Hospital), Jaruwan Kongkit (Vachira Phuket Hospital), Mongkontida Umphonsathien (Police General Hospital). **TURKEY:** Mehmet Akbulut (Firat University Medical Faculty Dept. of Cardiology), Gökhan Alici (Kosuyolu Traning and Research Hospital), Fahri Bayram (ErciYes University Medical Faculty Dept. of Endocrinology and Metabolism), Levent Hürkan Can (Ege University Medical Faculty Dept. of Cardiology), Ahmet Celik (Mersin University Medical Faculty Dept. of Cardiology), Ceyhun Ceyhan (Adnan Menderes University Medical Faculty Dept. of Cardiology), Fatma Yilmaz Coskun (Gaziantep University Medical Faculty Dept. of Cardiology), Mesut Demir (Cukurova University Medical Faculty Dept. of Cardiology), Sabri Demircan (Florence Nightingale Hospital), Volkan Dogan (Sitki Kocman University Medical Faculty Dept. of Cardiology), Emre Durakoglugil (Recep Tayyip Erdogan University Medical Faculty Dept. of Cardiology), İbrahim Etem Dural (Kocatepe University Medical Faculty Dept. of Cardiology), Omer Gedikli (Ondokuz Mayis University Medical Faculty Dept. of Cardiology), Aysa Hacioglu (ErciYes University Medical Faculty Dept. of Endocrinology and Metabolism), Muge Ildizli (Sultanbeyli State Hospital), Meral Kayikcioglu (Ege University Medical School, dept of Cardiology), Salih Kilic (Nizip State Hospital), Bahadir Kirilmaz (Onsekiz Mart University Medical Faculty), Merih Kutlu (Karadeniz Technical University Medical Faculty Dept. of Cardiology), Aytekin Oguz (Medeniyet University Medical Faculty Dept. of Internal Diseases), Oner Ozdogan (Tepecik Traning and Research Hospital), Ersel Onrat (Kocatepe University Medical Faculty Dept. of Cardiology), Savas Ozer (Karadeniz Technical University Medical Faculty Dept. of Cardiology), Tevfik Sabuncu (Harran University Medical Faculty Dept. of Endocrinology and Metabolism), Tayfun Sahin (Kocaeli University Medical Faculty Dept. of Cardiology), Fatih Sivri (Adnan Menderes University Medical Faculty Dept. of Cardiology), Alper Sonmez (Gulhane Training and Research Hospital Endocrinology and Metabolism Clinic), Ahmet Temizhan (Yuksek Ihtisas Training and Research Hospital, Dept. of Cardiology), Selim Topcu (Ataturk University Medical Faculty Dept. of Cardiology), Lale Tokgozoglu (Hacettepe University Medical Faculty Dept. of Cardiology), Abdullah Tuncez (Selcuk University Medical Faculty Dept. of Cardiology), Mirac Vural (Medeniyet University Medical Faculty Dept. of Internal Diseases), Mustafa Yenercag (Samsun Traning and Research Hospital), Dilek Yesilbursa (Uludag University Medical Faculty Dept. of Cardiology), Zerrin Yigit (Istanbul University Haseki Cardiology Institution), Aytul Belgi Yildirim (Akdeniz University Medical Faculty Dept. of Cardiology), Aylin Yildirir (Baskent University Medical Faculty Dept. of Cardiology), Mehmet Birhan Yilmaz (9 Eylül University Medical Faculty Dept. of Cardiology). **UNITED ARAB EMIRATES:** Bassam Atallah (Cleveland Clinic Abu Dhabi), Mahmoud Traina (Cleveland Clinic Abu Dhabi), Hani Sabbour (Cleveland Clinic Abu Dhabi), Dana Abdul Hay (Sheikh Khalifa Medical City), Neama Luqman (Sheikh Khalifa Medical City), Abubaker Elfatih (Sheikh Khalifa Medical City), Arshad Abdulrasheed (Sheikh Khalifa Medical City), Yosef Manla (Cleveland Clinic Abu Dhabi). **UNITED KINGDOM:** See Kwok (Manchester University NHS Foundation Trust). **URUGUAY:** Nicolas DellOca (GENYCO program, Comisión Nacional de Salud Cardiovascular). **UZBEKISTAN:** Rano B. Alieva (CAD and Atherosclerosis Dept., Republican Specialized Centre of Cardiology RSCC, Ministry of Health of Republic Uzbekistan, Tashkent), Khurshid G. Fozilov (Republican Specialized Centre of Cardiology RSCC, Ministry of Health of Republic Uzbekistan, Tashkent), Shavkat U. Hoshimov (CAD and Atherosclerosis Dept., RSCC, Ministry of Health of Republic Uzbekistan, Tashkent), Ulugbek I. Nizamov (CAD and Atherosclerosis Dept., RSCC, Ministry of Health of Republic Uzbekistan, Tashkent), Liliya E. Kan (CAD and Atherosclerosis Dept., RSCC, Ministry of Health of Republic Uzbekistan, Tashkent), Andrey R. Kim (CAD and Atherosclerosis Dept., RSCC, Ministry of Health of Republic Uzbekistan, Tashkent), Guzal J. Abdullaeva (Senior Researcher of the Molecular Genetic Research Group of RSCC, Ministry of Health of Republic Uzbekistan, Tashkent), Alisher A. Abdullaev (Center for Advanced Technologies, Ministry of Higher Education, Science and Innovation of Republic Uzbekistan, Tashkent). **VIETNAM:** Doan Loi Do (Vietnam National Heart Institute, Bach Mai Hospital, Hanoi; Dept. of Cardiology, Hanoi Medical University, Hanoi), Mai Ngoc Thi Nguyen (Vietnam National Heart Institute, Bach Mai Hospital, Hanoi; Dept. of Cardiology, Hanoi Medical University, Hanoi), Ngoc Thanh Kim (Vietnam National Heart Institute, Bach Mai Hospital, Hanoi; Dept. of Cardiology, Hanoi Medical University, Hanoi), Thanh Tung Le (Vietnam National Heart Institute, Bach Mai Hospital, Hanoi), Hong An Le (Vietnam National Heart Institute, Bach Mai Hospital, Hanoi).

# EAS Familial Hypercholesterolaemia Studies Collaboration (FHSC) Committees and National Lead Investigators

COORDINATING CENTRE

Kausik K. Ray (Lead Investigator), Amany Elshorbagy, Christophe A.T. Stevens, Kanika I. Dharmayat, Alexander R.M. Lyons, Antonio J. Vallejo-Vaz, Fotios Barkas (Imperial Centre for Cardiovascular Disease Prevention, Dept. of Primary Care and Public Health, School of Public Health, Imperial College London. London, United Kingdom).

EXECUTIVE COMMITTEE

Kausik K. Ray ([Lead Investigator] Imperial Centre for Cardiovascular Disease Prevention, Dept. of Primary Care and Public Health, School of Public Health, Imperial College London. London, United Kingdom), Alberico L. Catapano (University of Milan, Milan, Italy; IRCCS MultiMedica, Sesto San Giovanni, Milan, Italy), Tomas Freiberger (Centre for Cardiovascular Surgery and Transplantation, Brno, Czech Republic; and Medical Faculty, Masaryk University, Brno, Czech Republic), G. Kees Hovingh (Dept of vascular medicine, Academic Medical Centre, Amsterdam, The Netherlands), Pedro Mata (Fundación Hipercolesterolemia Familiar, Madrid, Spain), Frederick J. Raal (Carbohydrate and Lipid Metabolism Research Unit, Faculty of Health Sciences, University of the Witwatersrand, Johannesburg, South Africa), Raul D. Santos (Heart Institute InCor University of Sao Paulo and Hospital Israelita Albert Einstein, Sao Paulo, Brazil), Handrean Soran (Manchester University NHS Foundation Trust, Manchester, United Kingdom), Gerald F. Watts (School of Medicine, Faculty of Health and Medical Sciences, University of Western Australia, Perth, Australia; Lipid Disorders Clinic, Cardiometabolic Services, Dept. of Cardiology, Royal Perth Hospital, Perth, Australia).

STEERING COMMITTEE / NATIONAL LEAD INVESTIGATORS

ARGENTINA: Pablo Corral (FASTA University, School of Medicine, Pharmacology Dept., Mar del Plata, Argentina). AUSTRALIA: Gerald F. Watts (School of Medicine, Faculty of Health and Medical Sciences, University of Western Australia, Perth, Australia; Lipid Disorders Clinic, Cardiometabolic Services, Dept. of Cardiology, Royal Perth Hospital, Perth, Australia). AUSTRIA: Christoph J. Binder (Dept. of Laboratory Medicine, Medical University of Vienna, Vienna, Austria). BAHRAIN: Nasreen Al-Sayed (Gulf Medical & Diabetes Center, Bahrain). BELGIUM: Olivier S. Descamps (Department of Internal Medicine and Centre de Recherche Médicale de Jolimont, Pôle Hospitalier Jolimont, Réseau HELORA, Haine Saint-Paul and Department of Cardiology, Cliniques Universitaires Saint-Luc, Bruxelles, Belgium). BOSNIA AND HERZEGOVINA: Belma Pojskic (Cantonal Hospital Zenica, Zenica, Bosnia and Herzegovina). BRAZIL: Raul D. Santos (Heart Institute InCor University of Sao Paulo and Hospital Israelita Albert Einstein, Sao Paulo, Brazil). BULGARIA: Arman Postadzhiyan (Medical University of Sofia, Saint Anna University Hospital, Cardiology department, Sofia, Bulgaria). CANADA: Jacques Genest (McGill University, Montreal, Canada). CHILE: Rodrigo Alonso (Center for Advanced Metabolic Medicine and Nutrition, Santiago). CHINA: Jie Lin (Beijing Anzhen Hospital Capital Medical University, Beijing Institute of Heart Lung & Blood Vessel Diseases, Beijing, China), Luya Wang(Beijing Anzhen Hospital, Capital Medical University, Beijing Institute of Heart, Lung and Blood Vessel Diseases, Beijing, China). CHINA - HONG KONG: Brian Tomlinson (Faculty of Medicine, Macau University of Science and Technology, Macau, China). CROATIA: Željko Reiner (Dept. of Internal medicine, University Hospital Centre Zagreb, Zagreb). CYPRUS: Andrie G. Panayiotou (Cyprus International Institute for Environmental and Public Health, Cyprus University of Technology, Limassol, Cyprus). CZECH REPUBLIC:Tomas Freiberger (Centre for Cardiovascular Surgery and Transplantation, Brno, Czech Republic; and Medical Faculty, Masaryk University, Brno, Czech Republic). DENMARK: Børge G. Nordestgaard (Herlev and Gentofte Hospital, Copenhagen University Hospital, University of Copenhagen, Copenhagen, Denmark), Anne Tybjærg Hansen (Dept. of Clinical Biochemistry, Rigshospitalet, Copenhagen, Denmark; and Dept. of Clinical Medicine, Faculty of Health and Medical Sciences, University of Copenhagen, Copenhagen, Denmark). EGYPT: Ashraf Reda (Cardiology Dept., Faculty of medicine, Menoufia University, Menoufia, Egypt). ESTONIA: Margus Viigimaa (North Estonia Medical Centre, Tallinn University of Technology, Tallin). FRANCE: Eric Bruckert(Sorbonne University and Pitié Salpêtrière 3 Hospital APHP, Paris, France). GERMANY: Ulrich Laufs (Klinikund Poliklinik für Kardiologie, Universitätsklinikum Leipzig, Germany), Heribert Schunkert (Clinic for Heart and Circulatory Diseases, German Heart Centre Munich, Technical University Munich, Munich, Germany; DZHK16German Centre for Cardiovascular Research, Partner Site Munich Heart Alliance, Munich, Germany), Winfried März (DACH Society for the Prevention of Heart and Circulatory Diseases registered society, Hamburg, Germany; Dept. of Internal Medicine V Medical Faculty Mannheim, Heidelberg University, Mannheim, Germany; Klinisches Institut für Medizinische und Chemische Labordiagnostik, Medizinische Universität Graz,Graz, Austria; Synlab Akademie, Synlab Holding Deutschland GmbH, Mannheim und Augsburg, Germany).GREECE: Evangelos Liberopoulos (First Department of Propedeutic and Internal Medicine, School of Medicine, National and Kapodistrian University of Athens, Athens, Greece), Alexandros D. Tselepis (Atherothrombosis Research Centre, University of Ioannina, Ioannina, Greece). HUNGARY: György Paragh (Dept. of Internal Medicine, Faculty of Medicine, University of Debrecen, Debrecen, Hungary). INDIA: Tester F. Ashavaid (P.D.Hinduja Hospital and Medical Research Centre, Mahim, Mumbai, India). INDONESIA: Bambang Dwiputra(Department of Cardiology and Vascular Medicine, Universitas Indonesia - Harapan Kita National Cardiovascular Center, Jakarta, Indonesia). IRAN: Amirhossein Sahebkar (Mashhad Biotechnology Research Center, School of Medicine, Mashhad University of Medical Sciences, Mashhad). IRAQ: Mutaz Alkhnifsawi (Faculty of Medicine, University of Al Qadisiyah, Al Diwaniyah, Iraq). IRELAND: Vincent Maher (Advanced Lipid Management and Research Centre A.L.M.A.R. Tallaght University Hospital, Dublin, Ireland). ISRAEL: Ronen Durst (Cardiology Dept. and centre for cardiovascular precision medicine, HadassahHebrew University Medical Centre, Jerusalem, Israel). ITALY: Alberico L. Catapano (University of Milan,Milan, Italy; IRCCS MultiMedica, Sesto San Giovanni, Milan, Italy). JAPAN: Mariko Harada-Shiba(Osaka Medical and Pharmaceutical University, Osaka, Japan), Shizuya Yamashita (Rinku General Medical Centre, Osaka, Japan). KAZAKHSTAN: Kairat Davletov (Research Health Institute, Al Farabi Kazakh National University, Almaty, Kazakhstan). KUWAIT: Ahmad Al-Sarraf (Sabah Al Ahmad Cardiac Centre, Kuwait City, Kuwait). KYRGYZSTAN: Erkin Mirrakhimov (Kyrgyz State Medical Academy, Bishkek, Kyrgyzstan). LATVIA: Gustavs Latkovskis (Research Institute of Cardiology and Regenerative Medicine, Faculty ofMedicine, University of Latvia, Pauls Stradins Clinical University Hospital, Riga, Latvia). LEBANON: Marianne Abi Fadel (Laboratory of Biochemistry and Molecular Therapeutics, Faculty of Pharmacy, Saint Joseph University, Beirut, Lebanon). LITHUANIA: Zaneta Petrulioniene (Vilnius University Faculty of Medicine,Vilnius, Lithuania; Vilnius University Hospital Santaros Klinikos, Vilnius, Lithuania). MALAYSIA: Hapizah Nawawi (Institute of Pathology, Laboratory and Forensic Medicine I-P PerForM and Faculty of Medicine, Universiti Teknologi MARA UiTM, Sungai Buloh, Selangor, Malaysia). MALTA: Myra Tilney (Lipid Clinic, Mater Dei Hospital, Msida, Malta; Dept. of Medicine, Faculty of Medicine and Surgery, University of Malta, Msida, Malta). MEXICO: Carlos A. Aguilar-Salinas (Unidad de Investigación de Enfermedades Metabólicas, Instituto Nacional de Ciencias Médicas y Nutrición Salvador Zubirán, Mexico City, México; Tecnologico deMonterrey, Escuela de Medicina y Ciencias de la Salud, Monterrey, México), Alejandra Vázquez Cárdenas(Facultad de Medicina, Universidad Autónoma de Guadalajara, Mexico). NETHERLANDS: G. Kees Hovingh (Dept of vascular medicine, Academic Medical Centre, Amsterdam, The Netherlands), Erik S Stroes (dept of vascular medicine, Amsterdam university, Amsterdam), J Defesche (Department of Clinical Genetics, Amsterdam UMC Location AMC, Meibergdreef 9, 1105, AZ, Amsterdam), NIGERIA: Wilson Ehidiamen Sadoh (Dept. of Child Health, University of Benin Teaching Hospital, Benin City, Edo State, Nigeria). NORWAY: Martin P. Bogsrud (National Advisory Unit on FH, Oslo University Hospital, Oslo, Norway), KirstenB. Holven (National Advisory Unit on FH, Oslo University Hospital, Oslo, Norway). OMAN: Khalid Al Rasadi (Medical Research Center, Sultan Qaboos University, Muscat, Oman; Dept. of Biochemistry, College of Medicine& Health Science, Sultan Qaboos University, Muscat, Oman). PAKISTAN: Fouzia Sadiq (Directorate of Research, Shifa Tameer-e-Millat University, Islamabad, Pakistan). POLAND: Maciej Banach (Dept. of Preventive Cardiology and Lipidology, Medical University of Lodz, Poland; Polish Mother’s MemorialHospital Research Institute PMMHRI, Lodz, Poland; Cardiovascular Research Centre, University of ZielonaGora, Zielona Gora, Poland), Krzysztof Chlebus (1st Dept. of Cardiology Medical University of Gdansk, National Centre of Familial Hypercholesterolaemia in Gdańsk, Gdańsk, Poland). PORTUGAL: Mafalda Bourbon (Unidade de Investigação e Desenvolvimento, Grupo de Investigação Cardiovascular, Departamento de Promoçãoda Saúde e Prevenção de Doenças Não Transmissíveis, Instituto Nacional de Saúde Doutor Ricardo Jorge; BioISI– Biosystems & Integrative Sciences Institute, Faculty of Sciences, University of Lisbon, Lisbon, Portugal), DiogoCruz (Internal Medicine Department, Hospital de Cascais Dr. José de Almeida; Faculty of medicine, Lisbon University). ROMANIA: Dan Gaita (Institutul de Boli Cardiovasculare (IBCV - TIM), Universitateade Medicina si Farmacie Victor Babes din Timisoara, Romania). RUSSIA: Andrey V. Susekov (Academy for Postgraduate Medical Education, Faculty of Clinical Pharmacology and therapeutics, Ministry of Health, Russian Federation, Moscow, Russian Federation, Russia), Marat Ezhov (National Medical Research Centre of Cardiology of Ministry of Health of the Russian Federation, Moscow, Russia). SAUDI ARABIA: Fahad Alnouri (Cardiovascular Prevention Unit, Adult Cardiology Dept., Prince Sultan Cardiac Centre, Riyadh, Saudi Arabia), Khalid F. Alhabib (Dept. of Cardiac Sciences, King Fahad Cardiac Centre, College of Medicine, King Saud University, Riyadh, Saudi Arabia). SERBIA: Katarina Lalic (Faculty of Medicine University of Belgrade, Clinicfor Endocrinology, Diabetes and Metabolic Diseases, Belgrade, Serbia). SINGAPORE: Tavintharan17Subramaniam (Admiralty Medical Centre and Khoo Teck Puat Hospital, Yishun Health, Singapore). SLOVAKIA: Branislav Vohnout (Coordination Centre for Familial Hyperlipidemias, FOaZOS, Slovak Medical University in Bratislava, Dept. of Epidemiology, Medical Faculty, Comenius University, Bratislava, Slovakia). SLOVENIA: Urh Groselj (Dept. of Pediatric Endocrinology, Diabetes and Metabolism, UMC – University Children’s Hospital Ljubljana, Ljubljana, Slovenia; University of Ljubljana, Faculty of Medicine, Ljubljana, Slovenia). SOUTH AFRICA: Frederick J. Raal (Carbohydrate and Lipid Metabolism Research Unit, Faculty of Health Sciences, 4 University of the Witwatersrand, Johannesburg, South Africa), A.David Marais (Chemical Pathology, University of Cape Town Health Science Faculty, Cape Town, South Africa).SPAIN: Pedro Mata (Fundación Hipercolesterolemia Familiar, Madrid, Spain). SWITZERLAND: André R.Miserez (diagene Research Institute, Reinach, Switzerland; Faculty of Medicine, University of Basel, Basel, Switzerland; Swiss Society for Familial Forms of Hypercholesterolemia (SSFH), Reinach, Switzerland).TAIWAN: Ta-Chen Su (Dept.s of Internal Medicine and Environmental and Occupational Medicine, NationalTaiwan University Hospital, Taipei, Taiwan). THAILAND: Weerapan Khovidhunkit (Dept. of Medicine, Faculty of Medicine, Chulalongkorn University and King Chulalongkorn Memorial Hospital, Bangkok, Thailand). TURKIYE: Meral Kayikcioglu (Dept. of Cardiology, Ege University Medical School, Izmir, Turkiye).UKRAINE: Olena Mitchenko (Dyslipidaemia Dept., Institute of cardiology AMS, Kiev, Ukraine). UNITEDARAB EMIRATES: Wael Almahmeed (Heart and Vascular Institute, Cleveland Clinic Abu Dhabi, Abu Dhabi,UAE). UNITED KINGDOM: Handrean Soran (Manchester University NHS Foundation Trust, Manchester, United Kingdom). URUGUAY: Ximena Reyes (Programa Nacional de Detección Temprana y Atención de Hipercolesterolemia Familiar - GENYCO, Comisión Honoraria para la Salud Cardiovascular, Uruguay). UZBEKISTAN: Aleksandr B. Shek (Coronary Heart Disease and Atherosclerosis Dept., Republican Specialized Centre of Cardiology RSCC, Ministry of Health of Republic Uzbekistan, Tashkent, Uzbekistan). VENEZUELA: Marcos Miguel Lima-Martinez (Universidad de Oriente, Nucleo Bolívar, Ciudad Bolívar, Venezuela).VIETNAM: Thanh-Huong Truong (Faculty of Medicine, Phenikaa University, and Vietnam Atherosclerosis Society, Hanoi, Vietnam).

# Supplementary Tables

## Table S1. List of countries contributing data to the analysis of ASCVD

| **Variable** | **List of countries** |
| --- | --- |
| **Coronary artery disease** | ARG, AUS, AUT, BEL, BIH, BRA, CAN, CHL, DEU, DNK, ESP, EST, GBR, GRC, IDN, IND, IRQ, IRL, KAZ, JPN, LBN, LTU, LVA, MEX, MLT, MYS, NGA, NLD, NOR, OMN, POL, PRT, RUS, SGP, SVN, THA, TUR, TWN, UKR, URY, UZB, VEN and VNM |
| **Premature coronary artery disease** | ARE, ARG, AUS, AUT, BEL, BIH, BGR, CHL, CZE, DEU, DNK, ESP, EST, GBR, GRC, IDN, IND, IRQ, IRL, ITA, JPN, KAZ, LBN, LTU, LVA, MEX, MLT, MYS, NLD, NOR, OMN, PRT, RUS, SAU, SGP, SVN, THA, TUR, TWN, UKR, URY, UZB, VEN and VNM |
| **Stroke** | ARG, AUS, AUT, BEL, BIH, BRA, CHL, DEU, DNK, ESP, EST, GBR, GRC, IDN, IND, IRQ, IRL, JPN, KAZ, LBN, LTU, LVA, MEX, MLT, MYS, NGA, NLD, OMN, POL, PRT, RUS, SGP, SVN, THA, TUR, TWN, UKR, URY, UZB, VEN and VNM |
| **Peripheral artery disease** | ARG, AUS, AUT, BEL, BIH, BRA, DEU, DNK, ESP, EST, GRC, IDN, IND, IRQ, IRL, JPN, KAZ, LBN, LTU, LVA, MEX, MLT, MYS, OMN, POL, RUS, THA, TUR, TWN, UKR, URY, UZB, VEN and VNM |

ASCVD, atherosclerotic cardiovascular disease

ARG, Argentina; AUS, Australia; AUT, Austria; BEL, Belgium; BGR, Bulgaria; BIH, Bosnia; BRA, Brazil; CAN, Canada; CHL, Chile; CZE, Czech Republic; DEU, Germany; DNK, Denmark; ESP, Spain; EST, Estonia; GBR, Great Britain; GRC, Greece; IDN, Indonesia; IND, India; IRQ, Iraq; IRL, Ireland; ITA, Italy; JPN, Japan; KAZ, Kazakhstan; LBN, Lebanon; LTU, Lithuania; LVA, Latvia; MEX, Mexico; MLT, Malta; MYS, Malaysia; NGA, Nigeria; NLD, Netherlands; NOR, Norway; OMN, Oman; POL, Poland; PRT, Portugal; RUS, Russia; SAU, Saudi Arabia; SGP, Singapore; SVN, Slovenia; THA, Thailand; TUR, Turkey; TWN, Taiwan; UKR, Ukraine; URY, Uruguay; UZB, Uzbekistan; VEN, Venezuela; VNM, Vietnam.

## Table S2. List of countries included, and number of patients contributed to the present study by United Nations region

|  | **Children and adolescents** | **Adults** |
| --- | --- | --- |
| **Africa** | **0** | **57** |
| EGY | 0 | 52 |
| NGA | 0 | 5 |
| **Americas** | **292** | **2310** |
| ARG | 7 | 78 |
| BRA | 0 | 853 |
| CAN | 239 | 1011 |
| CHL | 6 | 48 |
| MEX | 19 | 189 |
| URY | 20 | 120 |
| VEN | 1 | 11 |
| **Asia** | **179** | **2823** |
| ARE | 0 | 20 |
| IDN | 0 | 5 |
| IND | 2 | 58 |
| IRQ | 1 | 26 |
| JPN | 6 | 240 |
| KAZ | 0 | 49 |
| LBN | 2 | 9 |
| MYS | 5 | 706 |
| OMN | 4 | 27 |
| SAU | 4 | 100 |
| SGP | 3 | 260 |
| THA | 1 | 230 |
| TUR | 0 | 443 |
| TWN | 0 | 230 |
| UZB | 2 | 144 |
| VNM | 23 | 44 |
| **Europe** | **5878** | **24071** |
| AUT | 126 | 231 |
| BEL | 54 | 642 |
| BGR | 4 | 122 |
| BIH | 0 | 9 |
| CZE | 200 | 1461 |
| DEU | 14 | 911 |
| DNK | 0 | 501 |
| ESP | 110 | 504 |
| EST | 0 | 4 |
| GBR | 0 | 7 |
| GRC | 363 | 922 |
| HRV | 1 | 0 |
| IRL | 1 | 17 |
| ITA | 913 | 1537 |
| LTU | 0 | 37 |
| LVA | 0 | 182 |
| MLT | 0 | 21 |
| NLD | 3526 | 14272 |
| NOR | 0 | 306 |
| POL | 46 | 1419 |
| PRT | 59 | 80 |
| RUS | 57 | 757 |
| SVN | 392 | 5 |
| UKR | 12 | 124 |
| **Oceania** | **52** | **236** |
| AUS | 52 | 236 |

ASCVD, atherosclerotic cardiovascular disease

ARE, United Arab Emirates; ARG, Argentina; AUS, Australia; AUT, Austria; BEL, Belgium; BGR, Bulgaria; BIH, Bosnia; BRA, Brazil; CAN, Canada; CHL, Chile; CZE, Czech Republic; DEU, Germany; DNK, Denmark; EGY, Egypt; ESP, Spain; EST, Estonia; GBR, Great Britain; GRC, Greece; HRV, Croatia; IDN, Indonesia; IND, India; IRQ, Iraq; IRL, Ireland; ITA, Italy; JPN, Japan; KAZ, Kazakhstan; LBN, Lebanon; LTU, Lithuania; LVA, Latvia; MEX, Mexico; MLT, Malta; MYS, Malaysia; NGA, Nigeria; NLD, Netherlands; NOR, Norway; OMN, Oman; POL, Poland; PRT, Portugal; RUS, Russia; SAU, Saudi Arabia; SGP, Singapore; SVN, Slovenia; THA, Thailand; TUR, Turkey; TWN, Taiwan; UKR, Ukraine; URY, Uruguay; UZB, Uzbekistan; VEN, Venezuela; VNM, Vietnam.

## Table S3. List of countries included, and number of patients contributed to the present study by World Bank income category

| **High income countries** | | |  | **Non-high income countries** | | |
| --- | --- | --- | --- | --- | --- | --- |
|  | **Children and adolescents**  **N = 6139** | **Adults**  **N = 25,121** |  |  | **Children and adolescents**  **N = 136** | **Adults**  **N = 4144** |
| ARE | 0 | 20 |  | ARG | 7 | 78 |
| AUS | 52 | 236 |  | BGR | 4 | 122 |
| AUT | 126 | 231 |  | BIH | 0 | 9 |
| BEL | 54 | 642 |  | BRA | 0 | 853 |
| CAN | 239 | 1011 |  | EGY | 0 | 52 |
| CHL | 6 | 48 |  | IDN | 0 | 5 |
| CZE | 200 | 1461 |  | IND | 2 | 58 |
| DEU | 14 | 911 |  | IRQ | 1 | 26 |
| DNK | 0 | 501 |  | KAZ | 0 | 49 |
| ESP | 110 | 504 |  | LBN | 2 | 9 |
| EST | 0 | 4 |  | MEX | 19 | 189 |
| GBR | 0 | 7 |  | MYS | 5 | 706 |
| GRC | 363 | 922 |  | NGA | 0 | 5 |
| HRV | 1 | 0 |  | RUS | 57 | 757 |
| IRL | 1 | 17 |  | THA | 1 | 230 |
| ITA | 913 | 1537 |  | TUR | 0 | 443 |
| JPN | 6 | 240 |  | TWN | 0 | 230 |
| LTU | 0 | 37 |  | UKR | 12 | 124 |
| LVA | 0 | 182 |  | UZB | 2 | 144 |
| MLT | 0 | 21 |  | VEN | 1 | 11 |
| NLD | 3526 | 14272 |  | VNM | 23 | 44 |
| NOR | 0 | 306 |  |  | | |
| OMN | 4 | 27 |  |  |  |  |
| POL | 46 | 1419 |  |  |  |  |
| PRT | 59 | 80 |  |  |  |  |
| SAU | 4 | 100 |  |  |  |  |
| SGP | 3 | 260 |  |  |  |  |
| SVN | 392 | 5 |  |  |  |  |
| URY | 20 | 120 |  |  |  |  |

ARE, United Arab Emirates; ARG, Argentina; AUS, Australia; AUT, Austria; BEL, Belgium; BGR, Bulgaria; BIH, Bosnia; BRA, Brazil; CAN, Canada; CHL, Chile; CZE, Czech Republic; DEU, Germany; DNK, Denmark; EGY, Egypt; ESP, Spain; EST, Estonia; GBR, Great Britain; GRC, Greece; HRV, Croatia; IDN, Indonesia; IND, India; IRQ, Iraq; IRL, Ireland; ITA, Italy; JPN, Japan; KAZ, Kazakhstan; LBN, Lebanon; LTU, Lithuania; LVA, Latvia; MEX, Mexico; MLT, Malta; MYS, Malaysia; NGA, Nigeria; NLD, Netherlands; NOR, Norway; OMN, Oman; POL, Poland; PRT, Portugal; RUS, Russia; SAU, Saudi Arabia; SGP, Singapore; SVN, Slovenia; THA, Thailand; TUR, Turkey; TWN, Taiwan; UKR, Ukraine; URY, Uruguay; UZB, Uzbekistan; VEN, Venezuela; VNM, Vietnam.

## Table S4. List of countries included and number of patients contributing to the present study by United Nations sub-region

|  | **Children and adolescents** | **Adults** |
| --- | --- | --- |
| **Central Asia** | **0** | **193** |
| KAZ | 0 | 49 |
| UZB | 0 | 144 |
| **Eastern Asia** | **6** | **470** |
| JPN | 6 | 240 |
| TWN | 0 | 230 |
| **Eastern Europe** | **319** | **3883** |
| BGR | 4 | 122 |
| CZE | 200 | 1461 |
| POL | 46 | 1419 |
| RUS | 57 | 757 |
| UKR | 12 | 124 |
| **Latin America and the Caribbean** | **55** | **1299** |
| ARG | 7 | 78 |
| BRA | 0 | 853 |
| CHL | 6 | 48 |
| MEX | 19 | 189 |
| URY | 20 | 120 |
| UZB | 2 | 0 |
| VEN | 1 | 11 |
| **Northern Africa and Western Asia** | **11** | **677** |
| ARE | 0 | 20 |
| EGY | 0 | 52 |
| IRQ | 1 | 26 |
| LBN | 2 | 9 |
| OMN | 4 | 27 |
| SAU | 4 | 100 |
| TUR | 0 | 443 |
| **Northern America** | **239** | **1011** |
| CAN | 239 | 1011 |
| **Northern Europe** | **1** | **1054** |
| DNK | 0 | 501 |
| EST | 0 | 4 |
| GBR | 0 | 7 |
| IRL | 1 | 17 |
| LTU | 0 | 37 |
| LVA | 0 | 182 |
| NOR | 0 | 306 |
| **Oceania** | **52** | **236** |
| AUS | 52 | 236 |
| **South-Eastern Asia** | **32** | **1245** |
| IDN | 0 | 5 |
| MYS | 5 | 706 |
| SGP | 3 | 260 |
| THA | 1 | 230 |
| VNM | 23 | 44 |
| **Southern Asia** | **2** | **58** |
| IND | 2 | 58 |
| **Southern Europe** | **1838** | **3078** |
| BIH | 0 | 9 |
| ESP | 110 | 504 |
| GRC | 363 | 922 |
| HRV | 1 | 0 |
| ITA | 913 | 1537 |
| MLT | 0 | 21 |
| PRT | 59 | 80 |
| SVN | 392 | 5 |
| **Sub-Saharan Africa** | **0** | **5** |
| NGA | 0 | 5 |
| **Western Europe** | **3720** | **16056** |
| AUT | 126 | 231 |
| BEL | 54 | 642 |
| DEU | 14 | 911 |
| NLD | 3526 | 14272 |

ARE, United Arab Emirates; ARG, Argentina; AUS, Australia; AUT, Austria; BEL, Belgium; BGR, Bulgaria; BIH, Bosnia; BRA, Brazil; CAN, Canada; CHL, Chile; CZE, Czech Republic; DEU, Germany; DNK, Denmark; EGY, Egypt; ESP, Spain; EST, Estonia; GBR, Great Britain; GRC, Greece; HRV, Croatia; IDN, Indonesia; IND, India; IRQ, Iraq; IRL, Ireland; ITA, Italy; JPN, Japan; KAZ, Kazakhstan; LBN, Lebanon; LTU, Lithuania; LVA, Latvia; MEX, Mexico; MLT, Malta; MYS, Malaysia; NGA, Nigeria; NLD, Netherlands; NOR, Norway; OMN, Oman; POL, Poland; PRT, Portugal; RUS, Russia; SAU, Saudi Arabia; SGP, Singapore; SVN, Slovenia; THA, Thailand; TUR, Turkey; TWN, Taiwan; UKR, Ukraine; URY, Uruguay; UZB, Uzbekistan; VEN, Venezuela; VNM, Vietnam.

## Table S5. Prevalence of different body weight categories by sex

|  | **Children and adolescents** | | **Adults** | |
| --- | --- | --- | --- | --- |
|  | | | | |
| **Global** | **Girls**  **N = 3163** | **Boys**  **N = 3112** | **Women**  **N = 15,750** | **Men**  **N = 13,508** |
| Underweight | 3.3% | 3.8% | 2.6% | 1.2% |
| Normal weight | 72.1% | 67.7% | 50.5% | 40.9% |
| Overweight | 17.5% | 18.1% | 29.7% | 42.4% |
| Obese | 7.1% | 10.4% | 17.2% | 15.4% |
|  | | | | |
| **Non-high income countries** | **Girls**  **N = 74** | **Boys**  **N = 62** | **Women**  **N = 2502** | **Men**  **N = 1825** |
| Underweight | 8.1% | 12.9% | 1.9% | 1.2% |
| Normal weight | 71.6% | 46.8% | 37.1% | 33.2% |
| Overweight | 10.8% | 24.2% | 36.1% | 44.5% |
| Obese | 9.5% | 16.1% | 24.9% | 21.2% |
| **High income countries** | **Girls**  **N = 3089** | **Boys**  **N = 3050** | **Women**  **N = 13,248** | **Men**  **N = 11,684** |
| Underweight | 3.1% | 3.6% | 2.7% | 1.2% |
| Normal weight | 72.1% | 68.1% | 53.0% | 42.1% |
| Overweight | 17.7% | 18.0% | 28.5% | 42.1% |
| Obese | 7.1% | 10.3% | 15.8% | 14.5% |

## Table S6. Prevalence and odds of ASCVD in underweight vs. normal weight adults with HeFH

|  | **N with condition/total N (%)** | | **P value^1^** |  | **OR (95% CI)^2^** | **P value** |
| --- | --- | --- | --- | --- | --- | --- |
|  | **Underweight** | **Normal weight** |  |  |  |  |
| **Coronary artery disease** | 29/455 (6.4%) | 1375/11,564 (11.0%) | <0.001 |  | 0.80 (0.51, 1.19) | 0.24 |
| **Premature coronary artery disease** | 17/503 (3.4%) | 816/11,068 (6.9%) | 0.002 |  | 0.72 (0.43, 1.18) | 0.19 |
| **Stroke** | 9/428 (2.1%) | 181/11,205 (1.6%) | 0.44 |  | 1.74 (0.86, 3.50) | 0.12 |
| **Peripheral artery disease** | 5/156 (3.2%) | 132/3251 (3.9%) | 0.66 |  | 1.03 (0.41,2.59) | 0.95 |

^1^Pearson’s Chi squared test.

^2^OR of having each condition, in underweight vs. normal weight adults, using logistic regression, adjusted for age and sex (Model 1).

ASCVD, atherosclerotic cardiovascular disease; HeFH, heterozygous familial hypercholesterolaemia

## Table S7. Age (years) at FH diagnosis in adults by body weight category in different United Nations sub-regions

|  | **Normal weight** | **Overweight** | **Obese** |
| --- | --- | --- | --- |
| Central Asia | 41 (34, 46)^1^ N = 27 | 45 (39, 50) N = 69 | 42 (37, 47) N = 48 |
| Eastern Asia | 50 (36, 57) N = 344 | 50 (37, 57) N = 109 | 44 (37, 56) N = 17 |
| **Eastern Europe** | **35 (23, 50) N = 1302** | **48 (36, 57.6) N = 1043** | **51 (41, 59) N = 651** |
| **Latin America and the Caribbean** | **43 (30.6, 55) N = 546** | **47 (37, 58) N = 481** | **49 (36, 60) N = 264** |
| **Northern Africa and Western Asia** | **40 (29, 50) N = 120** | **44 (35, 51) N = 257** | **45 (36, 52) N = 264** |
| **Northern America** | **41 (32, 52) N = 447** | **45 (37, 56) N = 373** | **47 (39, 55) N = 191** |
| **Northern Europe** | **50 (37, 60) N = 354** | **53 (42, 61) N = 431** | **53 (44, 62) N = 196** |
| **Oceania** | **38 (28, 51) N = 84** | **49 (39, 57) N = 88** | **51 (42, 57) N = 64** |
| Southern Asia | 45 (40, 52) N = 16 | 53 (49, 57) N = 35 | 50 (38, 52) N = 7 |
| South-Eastern Asia | 48 (33, 58) N = 475 | 48 (37, 56) N = 389 | 49 (37, 57) N = 201 |
| **Southern Europe** | **35 (23, 47) N = 508** | **41 (30, 51) N = 635** | **45 (31, 53) N = 342** |
| **Western Europe** | **40 (28, 53) N = 8510** | **49 (37, 61) N = 5296** | **49 (38, 60) N = 1935** |

^1^Data are median (25th, 75th percentiles) age in years. Regions with significantly older age at FH diagnosis in obese vs. normal weight categories are depicted in bold font.

## Table S8. The association of overweight and obesity with ASCVD in the total adult population from all world regions, using lower BMI cutoffs for the Asian population^1^

|  | **Model 1** | **Model 2** | **Model 3** |
| --- | --- | --- | --- |
| **Coronary artery disease** | | | |
| Overweight vs. normal weight | 1.62 (1.49, 1.76)  *p* <0.001 | 1.56 (1.42, 1.72) *p* <0.001 | 1.35 (1.22, 1.49) *p* <0.001 |
| Obese vs. normal weight | 3.07 (2.81, 3.35)  *p* <0.001 | 3.27 (2.96, 3.62) *p* <0.001 | 2.34 (2.09, 2.61) *p* <0.001 |
| **Premature coronary artery disease** | | | |
| Overweight vs. normal weight | 1.69 (1.53, 1.87)  *p* <0.001 | 1.50 (1.34, 1.68) *p* <0.001 | 1.28 (1.13, 1.46) *p* <0.001 |
| Obese vs. normal weight | 4.07 (3.67, 4.51)  *p* <0.001 | 3.82 (3.41, 4.28) *p* <0.001 | 3.01 (2.64, 3.42) *p* <0.001 |
| **Stroke** | | | |
| Overweight vs. normal weight | 1.33 (1.09, 1.63)  *p* = 0.005 | 1.33 (1.06, 1.68) *p* = 0.014 | 1.27 (1.00, 1.61) *p* = 0.052 |
| Obese vs. normal weight | 1.89 (1.52, 2.35)  *p <*0.001 | 1.90 (1.49, 2.43) *p <*0.001 | 1.48 (1.14, 1.94) *p =* 0.004 |
| **Peripheral artery disease** | | | |
| Overweight vs. normal weight | 1.20 (0.95, 1.52) *p =* 0.12 | 1.21 (0.94, 1.56) p=0.15 | 1.09 (0.83, 1.42) p=0.51 |
| Obese vs. normal weight | 1.31 (1.03, 1.66)  p = 0.026 | 1.48 (1.15, 1.92) p = 0.003 | 1.06 (0.80, 1.40) p = 0.72 |

^1^BMI categories are defined as follows: for patients from Asian countries (see Supplementary Table S2 for country list and N): Normal weight: <22 kg/m2; overweight: 22-<25 kg/m2; obese: ≥25 kg/m2; for all other patients, standard WHO cutoffs were used.

Data are odds ratios and 95% confidence intervals for the presence of different types of ASCVD in adults with HeFH and overweight or obesity vs. normal weight HeFH adults, using lower cutoffs for patients from Asia (see Methods section for details). Models are adjusted for the following variables: Model 1, age and sex; Model 2, age, sex, lipid-lowering medication, LDL-C, triglycerides, HDL-C and LDL-C*lipid-lowering medication interaction; Model 3, all Model 2 variables, plus diabetes, hypertension and smoking.

## Table S9. Association of overweight and obesity with ASCVD in adults with genetically confirmed FH^1^

| **Outcome variable** | **Overweight vs. normal weight** | **Obese vs. normal weight** |
| --- | --- | --- |
| Coronary artery disease  N with condition/N total: 1788/14,924 | 1.30 (1.14, 1.48)  *p* <0.001 | 1.98 (1.70, 2.31)  *p <*0.001 |
| Premature coronary artery disease  N with condition/N total: 909/12,699 | 1.32 (1.11, 1.55)  *p =* 0.001 | 2.27 (1.87, 2.75  p <0.001 |
| Stroke  N with condition/N total: 270/12,894 | 1.34 (1.00, 1.78)  *p =* 0.047 | 1.61 (1.14, 2.28)  *p =* 0.007 |
| Peripheral artery disease  N with condition/N total: 90/3251 | 0.97 (0.56, 1.68)  *p =* 0.91 | 1.63 (0.93, 2.85)  *p =* 0.090 |

^1^Data are odds ratios (95% CI) and p values from logistic regression analysis, adjusted for age, sex, lipid-lowering medication, LDL-C, HDL-C, triglycerides and LDL-C*lipid-lowering medication interaction (Model 2).

ASCVD, atherosclerotic cardiovascular disease

## Table S10. Association of overweight and obesity with ASCVD in different adult subgroups^1^

| **Stratification variable** | **Overweight vs. normal weight** | **Obese vs. normal weight** | **Overweight vs. normal weight** | **Obese vs. normal weight** | | **P- interaction** |
| --- | --- | --- | --- | --- | --- | --- |
| **Age group** | **≤47 years** | | **>47 years** | | |  |
| Coronary artery disease | 1.56 (1.28, 1.92)  p <0.001 | 2.88 (2.28, 3.63)  p <0.001 | 1.37 (1.23, 1.53)  p <0.001 | | 1.97 (1.73, 2.24)  p <0.001 | 0.005 |
| Stroke | 2.02 (1.05, 4.02)  *p =* 0.039 | 2.04 (0.92, 4.47)  *p =* 0.074 | 1.20 (0.94, 1.53)  *p =* 0.14 | | 1.49 (1.12, 1.97)  *p =* 0.005 | 0.23 |
| Peripheral artery disease | 1.34 (0.75, 2.44)  *p =* 0.32 | 1.89 (1.02, 3.53)  *p =* 0.044 | 1.11 (0.83, 1.49  *p =* 0.51 | | 1.66 (1.22, 2.28  p <0.001 | 0.95 |
| **Sex** | **Women** | | **Men** | | |  |
| Coronary artery disease | 1.45 (1.26, 1.68)  *p<*0.001 | 2.01 (1.71, 2.35)  *p<*0.001 | 1.52 (1.34, 1.73)  *p<*0.001 | 2.71 (2.31, 3.18)  *p<*0.001 | | 0.10 |
| Premature coronary artery disease | 1.47(1.22, 1.79)  p <0.001 | 2.07(1.68, 2.54  p <0.001 | 1.43(1.23, 1.67)  p <0.001 | 2.42(2.01, 2.90)  p <0.001 | | 0.58 |
| Stroke | 1.36(1.00, 1.86)  *p =* 0.049 | 1.42(0.99, 2.01)  *p =* 0.053 | 1.26(0.90, 1.77)  *p =* 0.24 | 1.83(1.23, 2.71)  *p =* 0.003 | | 0.93 |
| Peripheral artery disease | 1.38 (0.98, 1.97)  *p =* 0.069 | 1.64 (1.12, 2.40)  *p =* 0.010 | 1.07 (0.72, 1.60)  *p =* 0.71 | 2.00 (1.33, 3.03)  p <0.001 | | 0.51 |
| **Index status** | **Index** | | **Non-index** | | |  |
| Coronary artery disease | 1.30 (1.10, 1.53)  *p* = 0.002 | 1.94 (1.60, 2.35)  *p* <0.001 | 1.26 (1.09, 1.47)  *p* <0.002 | 1.80 (1.49, 2.17)  *p* <0.001 | | 0.50 |
| Premature coronary artery disease | 1.13 (0.92, 1.39)  *p* = 0.24 | 1.65 (1.31, 2.09)  *p* <0.001 | 1.26 (1.02, 1.55)  *p* = 0.029 | 2.00 (1.55, 2.57)  *p* <0.001 | | 0.86 |
| Stroke | 1.15 (0.78, 1.70)  *p* <0.5 | 1.44 (0.93, 2.22)  *p* = 0.10 | 1.38 (1.01, 1.91)  *p* = 0.046 | 1.60 (1.07, 2.38)  *p* = 0.021 | | 0.68 |
| Peripheral artery disease | - 1. (0.72, 1.41)   *p* = 0.96 | 1.50 (1.05, 2.15)  *p* = 0.024 | 2.05 (0.84, 5.53)  *p* = 0.13 | 5.02 (2.10, 13.4)  *p* <0.001 | | 0.060 |

^1^Data are odds ratios (95% CI) and p values from logistic regression analysis, adjusted for age, sex, lipid-lowering medication, LDL-C, HDL-C, triglycerides and LDL-C*lipid-lowering medication interaction (Model 2). Age groups were based on median split.

ASCVD, atherosclerotic cardiovascular disease

# Supplementary Figures

## Supplementary Figure S1.


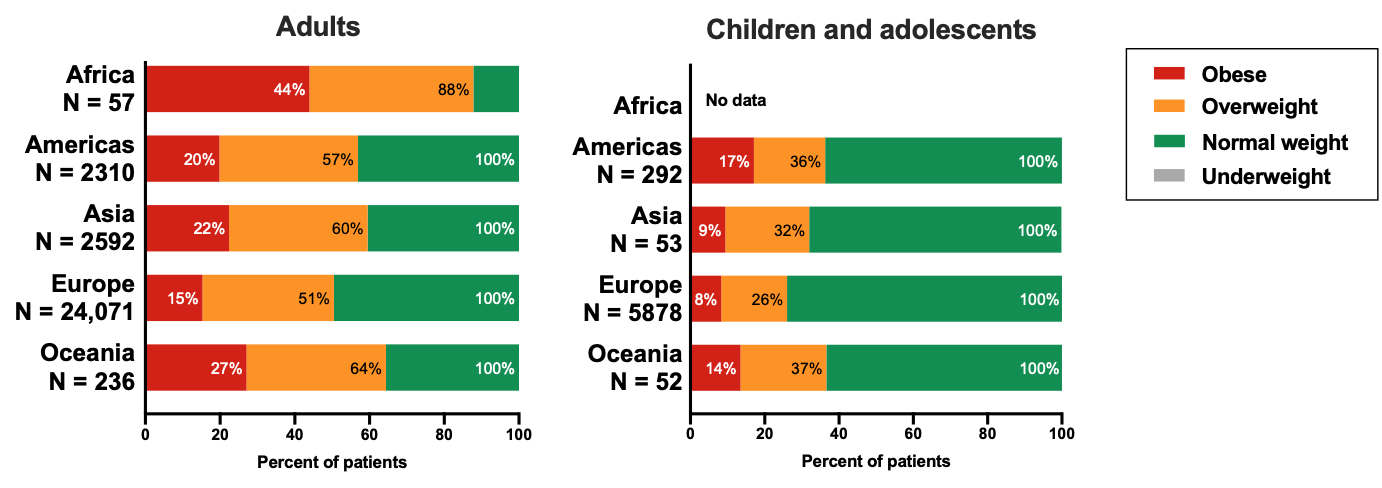

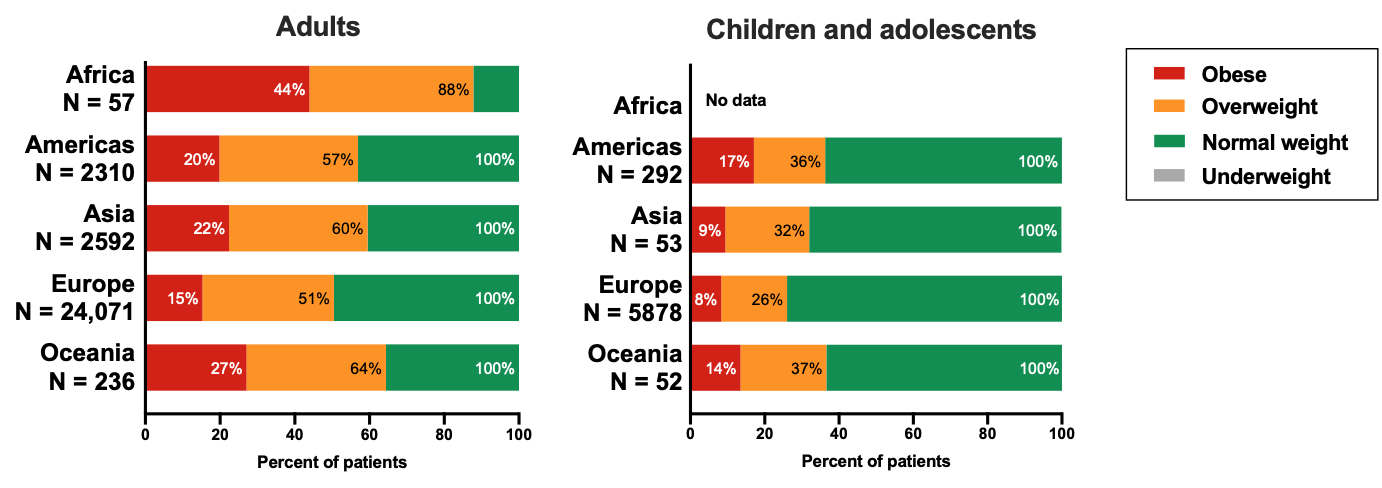


Figure S1. Prevalence of underweight, normal weight, overweight and obesity in people with heterozygous familial hypercholesterolemia by United Nations region. Cumulative percentages are depicted. Adults were aged 18 years or older; children and adolescents were aged 5-<18 years. Weight categories were defined by WHO body mass index cut-offs (see Methods section for details).

## Supplementary Figure S2.


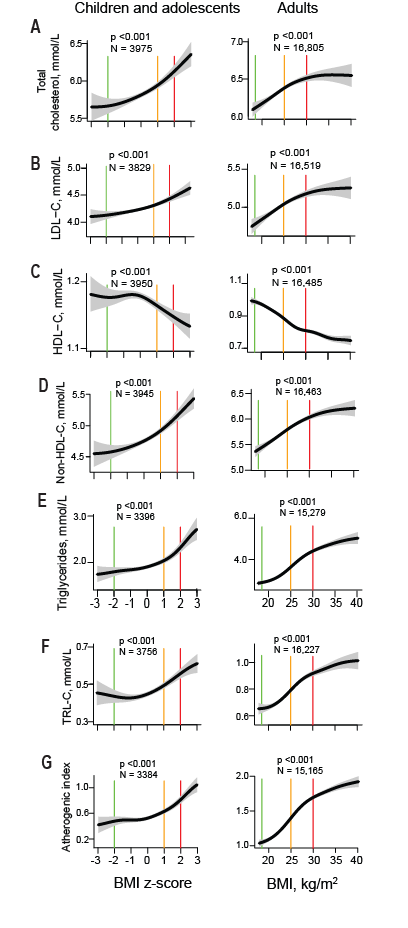


Figure S2. Sensitivity analysis in the subgroup with genetically confirmed FH. Estimated means and 95% confidence intervals of different plasma lipid fractions by BMI z-score (in children and adolescents (aged 5-<18 years; left panels) and adults (aged 18 years or older; right panels) with adjustment for age, sex and use of lipid-lowering medication. The green, yellow and red lines mark the beginning of the normal weight, overweight and obese BMI ranges respectively. The lowest and highest 1% of the independent variable are not shown. Note the different scales on the Y-axes. BMI, body mass index; TRL-C, triglyceride-rich lipoprotein cholesterol.
